# *Fusarium verticillioides* genetics contribute to variability in fumonisin risk in maize

**DOI:** 10.3389/fmicb.2026.1713439

**Published:** 2026-03-03

**Authors:** Joseph Opoku, Mark Busman, Lina Castano-Duque, Robert H. Proctor, Hye-Seon Kim, Martha M. Vaughan

**Affiliations:** 1 USDA, Agricultural Research Service, National Center for Agricultural Utilization Research, Mycotoxin Prevention and Applied Microbiology Research Unit, Peoria, IL, United States; 2 USDA, Agricultural Research Service, Southern Regional Research Center, Food and Feed Safety Research Unit, New Orleans, LA, United States

**Keywords:** disease modeling, fumonisins, *Fusarium verticillioides*, maize, mycotoxins, risk index

## Abstract

*Fusarium verticillioides* is a major fungal pathogen of maize and a primary cause of contamination of kernels with fumonisins—mycotoxins that threaten food safety and animal health. This study examined the influence of genetic diversity of *F. verticillioides* on the development of a fumonisin risk index. To do this, the effect of temperature (10–40 °C) on growth as assessed by ergosterol levels and fumonisin production in the fungus was assessed by liquid chromatography-mass spectrometry analysis, and the resulting data were subjected to a battery of analyses, including least squares means, Baranyi and Ratkowsky analyses. Although there was considerable variation among strains, the general trend was that growth of *F. verticillioides* occurred over a broader range of temperatures (15–35 °C) than fumonisin production (optimal at 20–30 °C). Growth and production were positively correlated (R^2^ = 0.524 overall; R^2^ = 0.78 at 30 °C), although the strength of this relationship varied with temperature. Production of the four major B-series fumonisin analogs (FB_1_–FB_4_) varied among strains, but for all strains the ratio of FB_1_–FB_2_ tended to increase with increasing temperature. These results demonstrate that fumonisin risk is shaped by a complex interplay of strain genetics and environmental conditions. The strain-dependent differences in growth kinetics, toxin production, and analog composition underscore the need for risk indices that integrate both environmental and genetic parameters to improve predictive models for mycotoxin contamination and targeted strategies to limit contamination during maize production and storage.

## 1 Introduction

Mycotoxins are toxic secondary metabolites produced by fungal pathogens that infect and contaminate cereal grains worldwide. Mycotoxin contamination of grains represents a significant threat to food safety, animal health, and economic gain across the entire grain value chain. Maize (*Zea mays* L.) production is particularly affected because crops are susceptible to the three major classes of mycotoxins (aflatoxins, fumonisins, and trichothecenes), and most of U.S. maize produced is used as food or feed.

While aflatoxins have greater toxicity and are considered highly carcinogenic, fumonisins are more prevalent contaminants in maize. According to a North American mycotoxin survey conducted by Dutch State Mines-Firmenich (DSM-Firmenich) in 2024, fumonisins were detected in 68% of sampled maize kernels. Fumonisins are a class of fungal metabolites synthesized via the polyketide pathway into structural analogs of sphingoid bases that inhibit ceramide synthase and disrupt sphingolipid metabolism, a mechanism that underlies their toxic effects across species (Voss et al., 2007; Wang et al., 1991; Lanubile et al., 2025). Of the many fumonisin analogs, based on their chemical structures, B series fumonisins are the most common contaminants and thus have been most intensively investigated for their toxicity. Fumonisin B_1_ (FB_1_) has been shown to cause a variety of negative health effects in horses, swine and laboratory rodents, and is correlated with human diseases and stunting humans. In livestock, FB_1_ has been causally linked to equine leukoencephalomalacia and porcine pulmonary edema. In humans, FB_1_ is classified as a “possible carcinogenic metabolite” and has been epidemiologically associated with esophageal cancer in high-exposure regions (Voss et al., 2007; Lanubile et al., 2025; Marasas, 2001). Additionally, consumption of FB1 contaminated maize has been linked to neural tube birth defects (Missmer et al., 2006; Waes et al., 2005).

Fumonisins are produced by multiple *Fusarium* species, including the maize pathogens *Fusarium verticillioides* (Sacc.) Nirenberg and *F. proliferatum* (Matsush.) Nirenberg (Rheeder et al., 2002; Proctor et al., 2013). However, *F. verticillioides* is considered the primary etiological agent responsible for fumonisin contamination of U.S. maize (Blacutt et al., 2018; Munkvold and Desjardins, 1997). *F. verticillioides* is a globally prevalent pathogen of maize, capable of colonizing roots, stems, and ears, often without causing visible symptoms (Marasas, 2001; Yates et al., 2003). The epidemiology of *F. verticillioides* and fumonisin contamination in maize is influenced by a complex interplay of factors, encompassing environmental conditions, including temperature, humidity and agronomic practices, as well as inoculum source (seedborne, crop residues, insect mediation via vectoring and wounding), and host physiology (Jacquat et al., 2025; Munkvold, 2003; Munkvold et al., 1999, 1997b; Miller, 2001; Opoku et al., 2019). Generally, Fusarium ear rot and fumonisin contamination of maize are thought to occur when moderate to warm temperatures coincide with maize silking, and contamination is typically exasperated by insect damage (Munkvold et al., 1997a; de la Campa et al., 2005; Stafstrom et al., 2025). Nevertheless, the environmental conditions interact directly or indirectly with host phenological stages and pathogen development, making it difficult to accurately assess risk (de la Campa et al., 2005; Wu, 2007). Additionally, within-species genetic variability may affect risk assessment outcomes. Thus, due to underlying genetic variability, *F. verticillioides* variants may respond differently to identical environmental conditions making it imperative to investigate how these differences impact predictive accuracy.

Due to food safety concerns and necessary mitigation strategies associated with fumonisins and other classes of mycotoxins, contamination causes significant economic losses to farmers and other stakeholders across the production and distribution chain. These losses arise from several interconnected factors, including yield reductions, grain quality downgrades, price penalties, mitigation costs, and trade restrictions that require stringent monitoring (Jacquat et al., 2025; Wu, 2007). To circumvent these losses, maize producers require reliable information and forecasting systems, so that they can make informed and timely decisions to deploy intervention strategies needed to minimize the health and economic impacts associated with mycotoxin contamination.

Recently, significant efforts have been made to provide U.S. corn growers with AI powered mycotoxin predictive modeling tools. However, the models developed are only suitable to predict contamination levels within limited regions for which historical mycotoxin data was available (Castano-Duque et al., 2023; Branstad-Spates et al., 2024; Castano-Duque et al., 2025). Therefore, models currently only exist for predictions of aflatoxin and fumonisin contamination in grain produced in Iowa (Branstad-Spates et al., 2024), Illinois (Castano-Duque et al., 2023) and Texas (Castano-Duque et al., 2025). However, the balanced accuracy, accounting for the rare event of high fumonisin levels, was only 50%, and 85% for fumonisin in Iowa and Illinois models, while for aflatoxin in Texas was 73%. The Iowa and Illinois-models do not include engineer input features that describe the relationship of temperature, *Fusarium* growth, and toxin production (Branstad-Spates et al., 2024); whereas the Texas model includes these mathematical relationships as input features (Castano-Duque et al., 2025).

The accuracy of the predictive models is contingent on the amount and quality of data that can be incorporated from the complex interacting factors of the disease triangle or the interlaced mycotoxin contamination triangle. Information of these factors can include host genotype (resistant or susceptible), environmental conditions (agronomic practices, soil type, insect damage Dowd et al., 2004, historical and predicted weather conditions), timing/host phenology, and lastly pathogen genotype and abundance. Laboratory studies providing knowledge regarding how the fungi grow and, importantly, synthesize mycotoxins in response to growth conditions can be assembled to produce a “risk index”. Here, we define a fumonisin risk index as a quantitative representation of how specific environmental or biological conditions influence the likelihood and magnitude of fumonisin production, which can be incorporated as an engineered feature to improve predictive model performance. Aflatoxin risk indexes have been incorporated into aflatoxin predictive models as engineered input features (Castano-Duque et al., 2022). Usage of these engineered features has been shown to increase the predictive model specific predictive accuracy by 22%, sensitivity by 28%, and specificity by 14% when compared to only-weather driven models (Castano-Duque et al., 2025). While aflatoxin risk indices have benefited predictive models for aflatoxin contamination, risk indices have not been incorporated into predictive models for fumonisin contamination in U.S. corn.

Several laboratory studies have examined the effects of temperature and water activity (a_w_) on *F. verticillioides* and *F. proliferatum* growth and fumonisin production (de la Campa et al., 2005; Stafstrom et al., 2025). However, results from these previous studies are inconsistent, possibly due to different methodologies used or intraspecific genetic differences among isolates. Clear differences exist in response to the abiotic factors between the two species (Samapundo et al., 2005), but little is known about how genetic diversity within species influences their responses and downstream fumonisin risk. Furthermore, regional specific differences in fumonisin producing fungal species and their effects on fumonisin risk have not been explored. Advancements in genome sequencing have begun to reveal that both species include considerable genetic variability (Degradi et al., 2024; Hudson et al., 2024; Niehaus et al., 2017). But the extent of the variability is unknown, and whether any of the variability is associated with host or geographic origin or variation in fumonisin production is not yet clear.

To fill the knowledge gaps and develop more pertinent fumonisin risk indexes needed to enhance the predictive power of fumonisin risk models, we revisited the effect of temperature on *F. verticillioides* growth and fumonisin production. Although past studies have demonstrated the importance of a_w_, it is currently not feasible to accurately incorporate a_w_ values into predictive models due to the variability in field measurements which are highly dependent on specific environmental conditions and microclimates (substrate, plant phenology and weather); therefore, variable a_w_ levels were not evaluated herein. Driven by the hypothesis that genetic diversity within *F. verticillioides* contributes to variability in temperature responses and that risk indexes derived from isolates spanning this diversity would provide greater predictive power, a phylogenetic comparison was initially made using 19 *F. verticillioides* strains and this knowledge was used to select five diverse isolates to evaluate growth and fumonisin production. The results were then used to compare strains and assess the potential influence of genetic diversity on a fumonisin risk index for *F. verticillioides*.

## 2 Methods

### 2.1 Evaluation of *F. verticillioides* diversity and isolate selection

Strains used for this study were selected from a collection of 19 strains (Supplementary Table 1). Whole genome sequence data were generated using a MiSeq-Illumina instrument at USDA ARS National Center for Agricultural Utilization Research in Peoria, Illinois. Prior to genome sequencing, each *F. verticillioides* strain was reisolated from single conidium and grown for 2–5 days on GYP agar medium (2% glucose, 2% peptone, 0.3% yeast extract, and 1.5% agar) amended with chloramphenicol (10 μg/mL). This was to suppress the growth of any extraneous bacteria contaminants to reduce competition, making it easier to obtain pure for downstream processes. A plug from the edge of the resulting culture was transferred to liquid GYP medium and incubated in the dark for 2–3 days at 28 °C with shaking. The resulting mycelia were harvested by filtration, lyophilized, and ground to a powder. Genomic DNA was extracted from ground mycelia using the DNeasy Plant Mini Kit [QIAGEN (Aarhus, Denmark)]. Sequencing libraries were prepared from the genomic DNA using the Nextera XT DNA Library Preparation Kit (New England BioLabs, Ipswich, MA, USA). The quality and quantity of the resulting libraries were confirmed using TapeStation (Agilent, Santa Clara, CA, USA) and Qubit Fluorometric Quantification (Thermo Fisher Scientific, Waltham, MA, USA) protocols, respectively. Sequence reads generated with the MiSeq instrument were imported into QIAGEN (Aarhus, Denmark) and screened against genome sequences of 84 bacterial species to detect and remove contaminating DNA. This step was included as a quality-control step to ensure that only fungal sequences were retained for assembly and downstream analyses. Reads were then trimmed to remove low-quality bases and then assembled using the following parameter settings in CLC (CLC bio, Cambridge, MA, USA): word size = 20; bubble size = 50; minimum contig length = 500; auto-detect paired distances = checked; and perform scaffolding = checked).

Full-length (exons and introns) of 52 housekeeping gene sequences (Supplementary Table 2) were retrieved from assembled genome sequences using the BLASTn function in CLC (CLC bio, Cambridge, MA, USA). The retrieved sequences for each gene were aligned using the MUSCLE function in the program MEGA 7 (Kumar et al., 2016) , and each alignment was subjected separately to maximum likelihood tree inference using the program IQ-Tree version 1.6.12 (Nguyen et al., 2015) to determine the best substitution model. The alignments were then concatenated with SequenceMatrix (Vaidya et al., 2011) and the concatenated alignments were subjected to maximum likelihood tree inference using IQ-Tree with partitions to ensure the best substitution model was used for each gene. Sequences from the closely related species *Fusarium coicis* (strain NRRL 66233) were used as the outgroup in the phylogenetic analysis.

Culture preparation and experimental design. Five strains representing a range of genetic diversity of *F. verticillioides* were selected for growth and fumonisin production analyses: NRRL 66786, NRRL 66476, NRRL 66787, NRRL 66790, and ISU 180. Suspensions of conidia were prepared from 7-day-old cultures grown on V8 juice agar (200 mL V-8 Juice, 800 mL distilled water, 3 g CaCO_3_ and 20 g agar) flooded with 4 mL of sterile distilled water. The resulting suspension was adjusted to 1 × 104 conidia/mL water used to inoculate cracked corn kernel medium (2.5 g of cracked corn kernels and 1 mL distilled water) contained in a five-dram vial (total 378 vials). Vials were covered loosely with caps and autoclaved. After the medium was cooled, each vial was inoculated with 1 mL of the appropriate conidia suspension (or sterile water for controls). Vials containing water-inoculated cracked corn kernel medium were used as controls. These water-inoculated vials served as essential negative controls to confirm that any fungal growth or fumonisin detected originated solely from the introduced *F. verticillioides* strains. They ensured the substrate contained no background contamination, verified that fumonisins were absent prior to inoculation, and allowed us to distinguish temperature driven changes in the substrate from those caused by fungal metabolism. Thus, the controls were necessary to validate all treatment derived fumonisin measurements. The water activity was estimated at 0.99 using a AquaLab 4TE (Addium Inc., Pullman, WA, USA) water activity meter. The corn was saturated, and a thin layer of excess water was visible, but it did not cover the cracked kernels. These conditions were purposely chosen to evaluate the fungus under optimal toxin producing conditions. Caps were left loosely fitted to allow gas exchange.

Vials were divided into six treatment groups corresponding to the five strains and uninoculated control, with 63 vials per group. Each group was further subdivided into seven temperature treatments: 10, 15, 20, 25, 30, 35, and 40 °C (9 vials per temperature per strain). Temperature conditions were maintained by placing the vials in randomized design into temperature-controlled incubators without light. Samples were collected at 2-, 5-, and 8-days post inoculation. At each of these timepoints, three vials per temperature per strain (*n* = 3) were removed and stored at −20 °C until further analysis.

### 2.2 Chemical analysis

For compatibility with fumonisin analysis, growth of *F. verticillioides* strains was estimated using ergosterol levels. Extraction methods of cultures for fumonisin (Plattner, 1999) and ergosterol (Henderson et al., 2011) quantification have been described previously. Briefly, for the fumonisin analysis, 2 g of cracked corn kernel culture material were extracted with 10 mL 1:1 acetonitrile:water. After shaking for 2 h, the solvent was transferred to an autosampler vial. For ergosterol analysis, the same culture material, from which the residual acetonitrile:water was removed, was extracted again but with 10 mL ethyl acetate with shaking for 2 h. The resulting extract was transferred to an autosampler vial. Liquid chromatography-tandem mass spectrometry (LC-MS/MS) of the extracts for fumonisin analysis (Plattner, 1999) and for ergosterol analysis (Henderson et al., 2011) was done according to previously described methods. Briefly, 10 μL of acetonitrile:water extract was applied to a Kinetex (Phenomenex, Torrance, CA) XB-C18 column (50 mm length, 2.1 mm diameter, 2.6 μm particle size, 100A pore size). Chromatography was performed by a Shimadzu Nexera 40 Series UHPLC system (Shimadzu Scientific Instruments, Inc., Columbia, MD, USA), which consisted of a SIL-40 autosampler coupled to a LC-40 binary gradient pump. The CTO-40 column oven temperature was maintained at 50 °C. A continuous 0.4 mL/min gradient flow of methanol and water received the sample injection. Both the methanol and water mobile phases were modified to contain 0.2% acetic acid and follow a 20%−95% gradient over 5 min. Flow proceeded to a Triple Quad 3500 triple quadrupole mass spectrometer (ABSciex LLC, Framingham, MA) equipped with a heated (300 °C) electrospray ionization source and operated in positive ionization mode. Multiple reaction monitoring modes scanned for transitions distinctive for FB_1_ (722 to 334 and 722 to 352 *m/z*), FB_2_ (706 to 354 and 706 to 336 *m/z*), FB_3_ (706 to 334 and 706 to 352 *m/z*), and FB_4_ (690 to 320 and 690 to 338 *m/z*) in their positive-ion [M+H]^+^ forms. Quantitation for FB_1_, FB_2_, FB_3_, and FB_4_ relied on the transitions from 722 to 334, 706 to 336, 706 to 336, and 690 to 320 *m/z*, respectively. FB_2_ and FB_3_ were distinguished by differences in retention time. Control of the LC-MS/MS instrument and data processing was done with SCIEX Analyst 1.7.2 Software. The limit of quantification for the analytical method was 0.01 mg/L for FB_1_; levels below this limit were reported as “not detected”. For ergosterol, 10 μL of ethyl acetate extract was applied to a Kinetex (Phenomenex, Torrance, CA) XB-C18 column (50 mm length, 2.1 mm diameter, 2.6 μm particle size, 100A pore size). Chromatography was performed by a Shimadzu Model 40 UHPLC system. The column oven temperature was maintained at 50 °C. A continuous 0.4 mL/min gradient flow of methanol and water received the sample injection. Both the methanol and water mobile phases were modified to contain 0.2% acetic acid and follow a 20%−98% gradient over 5 min. Flow proceeded to a Triple Quad 3500 triple quadrupole mass spectrometer equipped with a heated (450 °C) atmospheric pressure chemical ionization source and operated in positive ionization mode. Multiple reaction monitoring modes scanned for transitions distinctive for ergosterol (379 to 69 and 379 to 159 m/z), in their positive-ion [M+H]^+^ forms. Quantitation for ergosterol relied on the transition from 379 to 69 *m/z*. The limit of quantification for the analytical method was 0.01 mg/L for ergosterol; levels below this limit were reported as “not detected”.

### 2.3 Data analysis of ergosterol and fumonisins

Ergosterol and total fumonisin content (levels of FB_1_, FB_2_, FB_3_, and FB_4_ combined) data were compared using JMP statistical software version 17.0.0. Levels of ergosterol, total fumonisin content, or individual fumonisin analogs were independently compared by performing a full factorial (5 x 7 x 3; 5 strains,7 temperatures, 3 time points), fit least squares means statistical analysis for ergosterol and total fumonisin. This method finds the best-fitting line or curve by ensuring the predictions are as close as possible to the actual numbers by minimizing the sum of squared difference between the observed and predicted values. The analysis also allowed for comprehensive evaluation of all possible combinations of factors to determine how each factor and their interactions influence significant differences in levels of ergosterol and total fumonisin content. Thus, significant differences between strains at different temperatures could be determined. An effects test was used to determine factors contributing to variable mean differences. Tukey's honestly significant difference (HSD) tests were performed by temperature and day to further distinguish differences between the individual means. The relationship between ergosterol and fumonisin levels was visualized by performing a regression analysis and determining the R^2^ values for all data points, or datapoints for individual temperatures.

Rate analysis of growth, mycotoxin production and determination of fumonisin risk index parameters: The rate of *F. verticillioides* growth and fumonisin accumulation at different temperatures over time was evaluated. Growth and mycotoxin production maximum rates (mumax) from the five strains of *F. verticillioides* were calculated by fitting ergosterol amount or toxin amount per day for each temperature regime by using the Baranyi growth model (Baranyi and Roberts, 1995) included in the R package, growthrates (Hall et al., 2014; R Core Team, 2017). We used this equation because Baranyi model incorporates a lag phase for growth, this is a parameter for the initial physiological state of the organism (Baranyi and Roberts, 1995). We used the fitted growth and toxin rate values to fit a second model to evaluate rate as a function of temperature by using the Ratkowsky equation (Ratkowsky and Reddy, 2017) in R. The Ratkowsky equation allowed to model the mathematical relationship of temperature and rate in order to determine parameters of growth and toxin production equations (Equation 1).


(1) $$\begin{array}{l} rate=\;{\left\{a*\left(t-tmin\right)\right\}}^{2}*{\left(1-\;e^{\left(b*\left(t-tmax\right)\right)}\right)}^{2} \end{array}$$

The Ratkowsky model was used because this mathematical equation uses the square root model, which is a popular tool in predictive microbiology for modeling how temperature affects microbial growth rates (Ratkowsky and Reddy, 2017). In this equation, “a” represents a constant related to the slope of the growth curve prior to optimum growth; “b” is a parameter that modulates the effect of high temperatures on growth, “tmin” is the minimum temperature where growth is predicted to happen, tmax is the maximum temperature where growth stops, t is the measured temperature. Ratkowsky growth equation has been successfully applied to dozens of bacterial strains, insects, animals and plants showing consistent performance across diverse datasets (Ratkowsky and Reddy, 2017; Ratkowsky et al., 1983; Zwietering et al., 1991; Shi et al., 2017; Dey et al., 2020), which validates the robustness of this model to fit growth equations of other organisms, such as fungi.

## 3 Results

### 3.1 *F. verticillioides* diversity and strain selection

**Figure 1:**
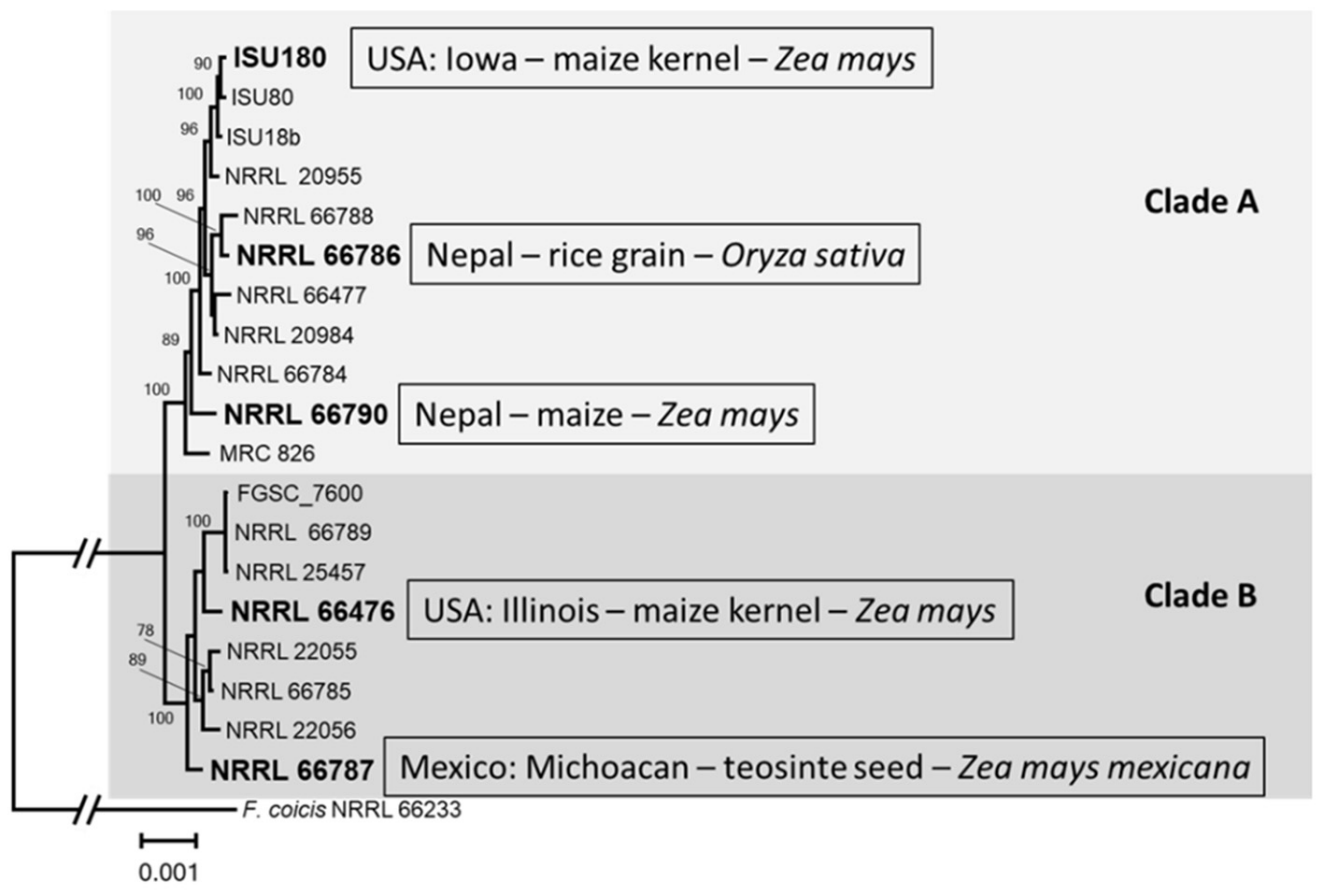
Maximum likelihood tree showing two major clades **(A, B)** of *F. verticillioides* strains. The tree was inferred from concatenated alignments of 52 housekeeping genes from loci across the *F. verticillioides* genome (Supplementary Table 2). Strains indicated in bold type were selected for the fumonisin risk index. Texts within boxes provide information on the origin of the strains. The tree was inferred using the program IQ-Tree, and numbers near the branches are bootstrap values based on 1,000 replicate trees. Sequences from *F. coicis* strain NRRL 66233 were used as an outgroup.

For the fumonisin risk index, we used a phylogenetic approach to select strains of *F. verticillioides* that included a range of genetic variation that exists within the species. To do this, we inferred a phylogenetic tree from full-length sequences of 52 housekeeping genes from a collection of 19 isolates of *F. verticillioides* recovered from diverse geographic regions (Supplementary Table 1). In the resulting tree, the *F. verticillioides* strains were resolved into two major clades (Clades A and B), each with high bootstrap support (Figure 1). Although the sampling of strains was limited, the tree indicated there is not a clear separation of strains by geographic origin. That is, one or more strains from Mexico, Nepal and the U.S. occurred in each of the two clades. For the risk index, we selected five strains that represented a wide range of the breadth of the phylogenetic diversity apparent in the tree (Figure 1). The selected strains were ISU 180, NRRL 66476, NRRL 66790, NRRL 66786, and NRRL 66787.

### 3.2 *F. verticillioides* biomass and total fumonisin

**Figure 2:**
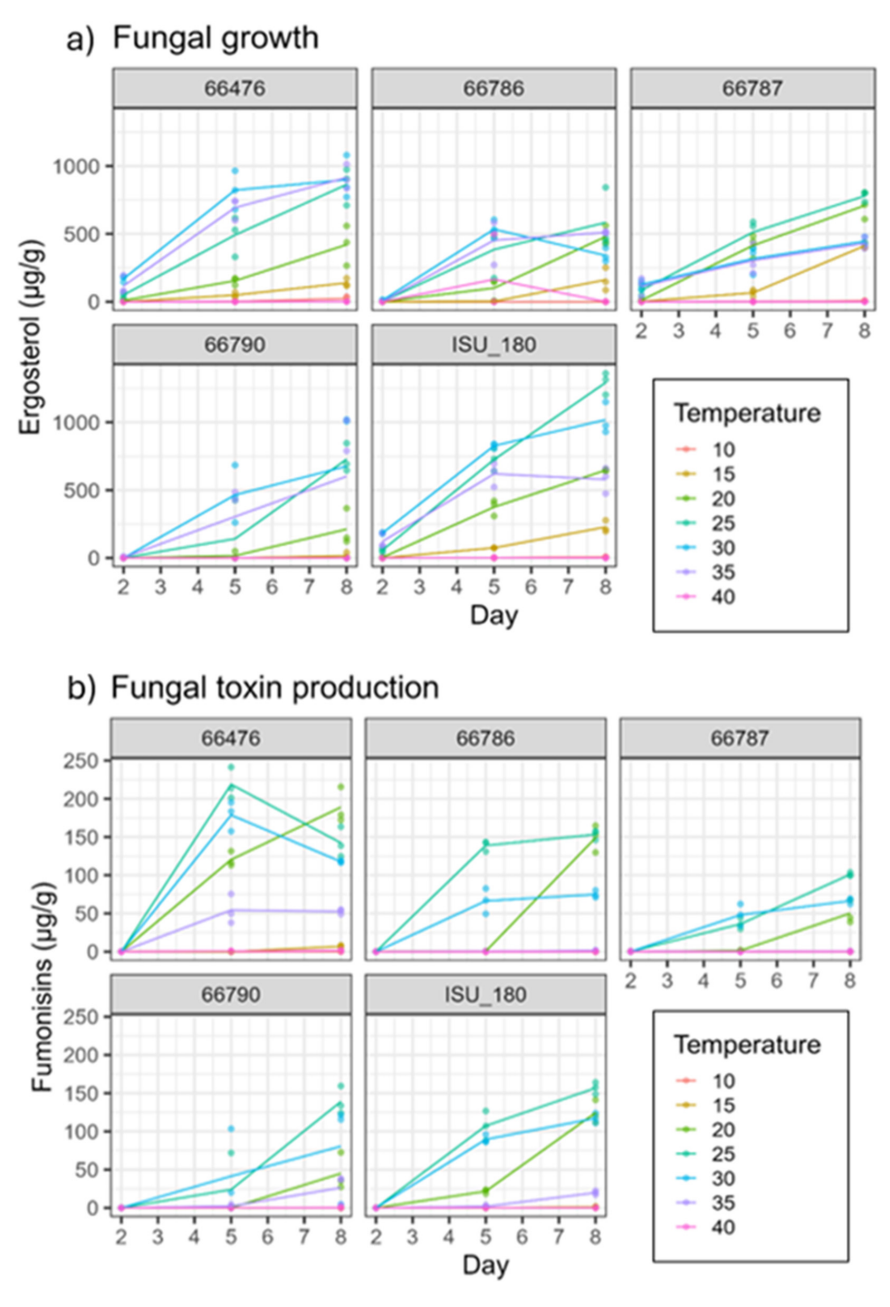
Ergosterol level [growth, **(a)**] and total fumonisin content **(b)** at 10–40 °C over time (day) by five *F. verticillioides* strains. Total fumonisin content was calculated by the sum of FB_1_, FB_2_, FB_3_, and FB_4_. Data from individual strains NRRL 66786, 66476, 66787, 66790, and ISU 180 are shown in separate panels. X-axis represents time (day), and Y-axis was measured ergosterol or total fumonisins content.

Preliminary analysis of data was done by generating graphs of fungal growth, assessed by ergosterol levels in cultures (μg/g), and total fumonisin B series content (i.e., FB_1_-FB_4_) at 5-degree increments from 10 to 40 °C at 2, 5 and 8 days of incubation (Figure 2). These data illustrated that the five strains primarily grew at 15 to 35 °C but showed only trace amounts of growth at 10 or 40 °C (Figure 2a and Supplementary Figure 1). There were two exceptions: 66790, which only showed trace amounts of growth at 15 °C, and 66786, which exhibited markedly high growth in one replicate culture at 40 °C on day 5. The optimal temperature range at which fumonisins were produced was narrower than the range for growth but strain dependent. All five strains produced fumonisins at 20–30 °C but only 66476, 66790, and ISU 180 produced fumonisins at 35 °C (Figure 2b). At 10 or 40 °C, all strains produced only trace amounts of fumonisins (Supplementary Figure 2).

**Figure 3:**
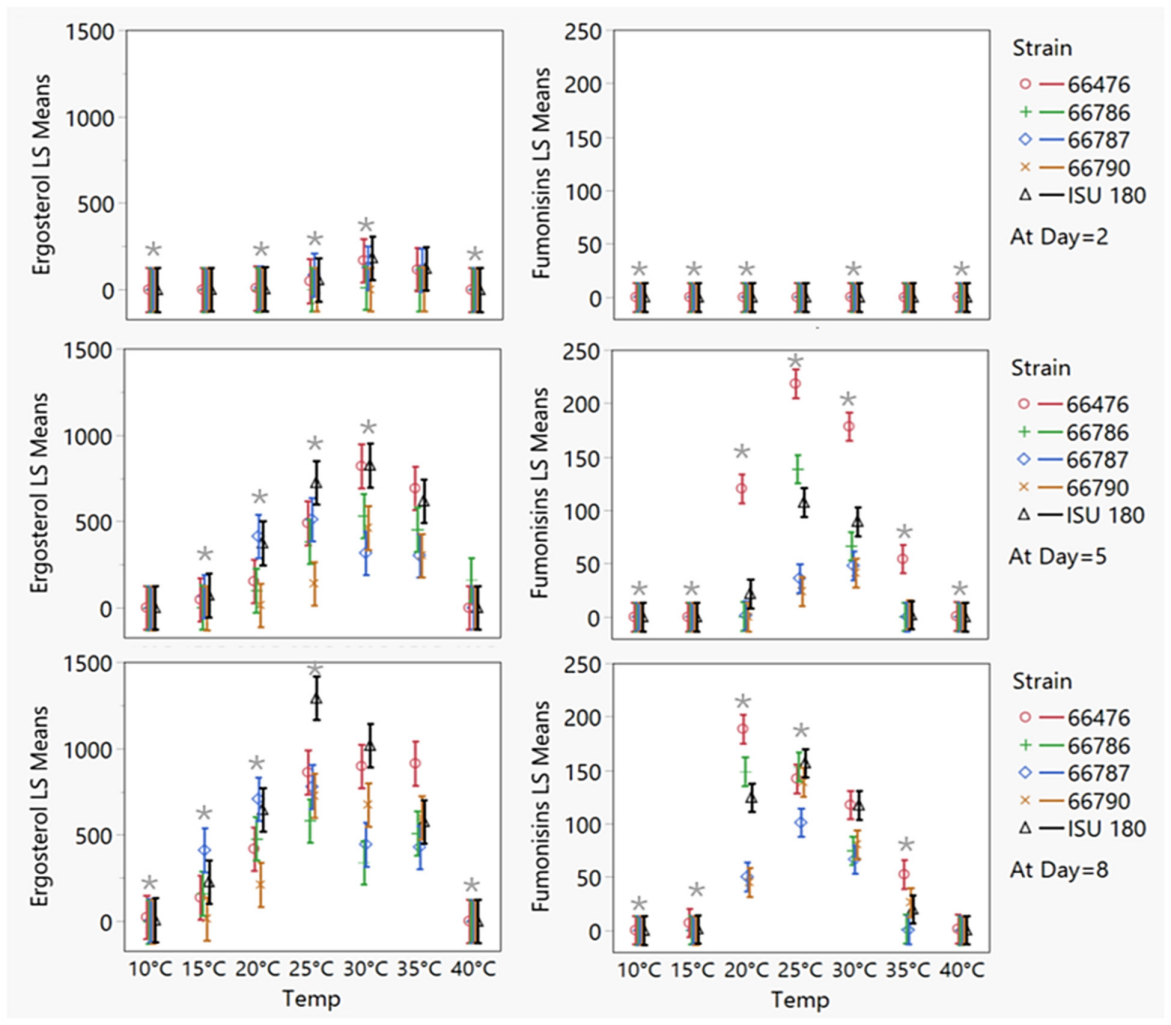
Least squares (LS) means plots for ergosterol (left) and total fumonisin content (right) as estimated by full factorial (5 x 7 x 3; 5 strains,7 temperatures, 3 time points), fit least squares means statistical analysis for individual *F. verticillioides* strains at different temperatures by timepoint (day). Bars represent confidence limits. Gray *denotes temperatures for which strain specific significant differences were determined by Tukey's honestly significant difference (HSD) tests (*P* < 0.05).

To evaluate the significance of the observed differences in ergosterol and total fumonisin, a 5 x 7 x 3 full factorial model (5 strains, 7 temperatures, and 3 time points) with fitted least squares means analysis was performed for ergosterol and total fumonisin content. The effects test identified all factors and interactions as significant contributors to differences (*P* < 0.0001), suggesting that ergosterol/growth and total fumonisin accumulation was time, temperature and strain dependent and that strains responded differently over time and to temperature. Mean ergosterol levels (growth) on day 8 were significantly greater than day 5, and levels on day 5 were significantly greater than day 2 according to Tukey's HSD test, *P* < 0.0001. Therefore, to visualize strain specific differences at different temperatures, least squares (LS) means were plotted by day (Figure 3). Furthermore, to evaluate which means contributed to differences among strains, Tukey's honestly significant difference (HSD) tests were performed for ergosterol and total fumonisin means, by temperature and day (Supplementary Figures 1, 2).

**Figure 4:**
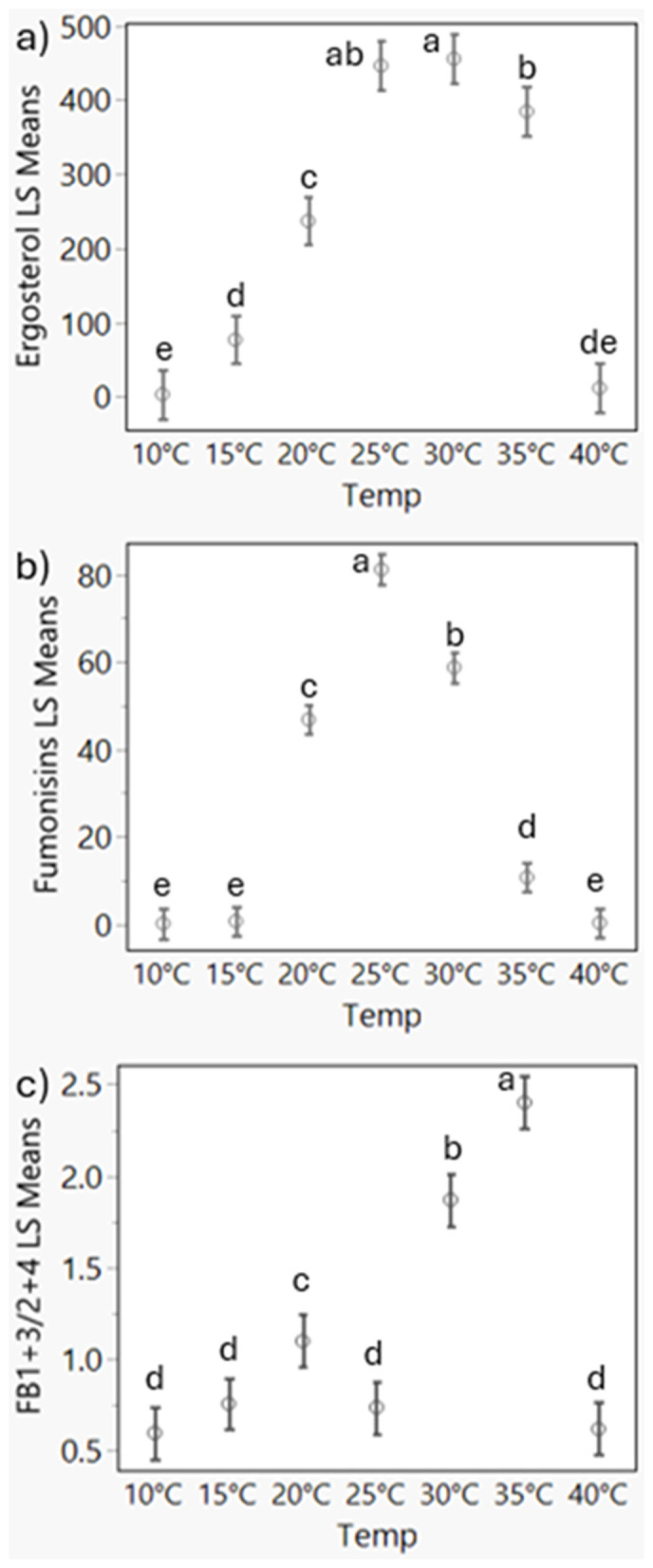
Least squares (LS) means plots for ergosterol **(a)**, fumonisins **(b)**, and ratio of FB_1_ + FB_3_/FB_2_ + FB_4_ **(c)** for all observations in relation to temperature. Fumonisins are the total of FB_1_, FB_2_, FB_3_, and FB_4_. The fumonisin ratio FB_1_ + FB_3_/FB_2_ + FB_4_ was calculated by dividing the sum of FB_1_ and FB_3_ by the sum of FB_2_ and FB_4_. Bars represent confidence limits. Means not sharing the same letters above the plots are significantly different as determined by Tukey's honestly significant difference (HSD) tests (*P* < 0.05).

Significant strain-specific differences in ergosterol/growth were observed for 14 of the 21 combinations evaluated (7 temperatures and 3 time points; Figure 3). Strains 66786 and 66790 appeared delayed in growth, as their ergosterol levels on day 2 at 20–30 °C were significantly lower than levels for other strains (Supplementary Figure 1, *P* < 0.03). However, by day 5 at 25 °C only 66790 had significantly lower ergosterol levels than ISU 180 (*P* = 0.01). Overall, strain ISU 180 had the highest ergosterol levels, but on day 2 at 10 and 20 °C and on day 8 at 15 °C, strain 66787 had significantly higher ergosterol levels than ISU 180. Temperature also contributed to significant differences in growth among strains (*P* < 0.0001). Considering all strains, growth was greatest at 25–35 °C, moderate at 20 °C, and minimal at the lowest (10–15 °C) and highest (40 °C) temperatures (Figure 4a). However, the optimal temperature for growth, represented by highest ergosterol levels, was strain dependent. ISU 180, 66786, 66787, and 66790 exhibited highest mean growth at 25 °C, while 66476 had highest growth ergosterol mean at 35 °C.

Nevertheless, only ISU 180 had significantly highest growth at 25 °C in comparison to other temperatures (Tukey's HSD, *P* < 0.01), while others had a range of temperatures. No significant difference was detected between 20–25 °C for 66787, 20–35 °C for 66786, 25–35 °C for 66476 and 66790. This suggests that individual strains had distinct optimal temperatures and ranges for growth.

Significant strain specific differences in total fumonisin were observed for 17 out of the 21 combinations evaluated (Figure 3). Significant differences in total fumonisin were detected among strains at most temperatures and days analyzed with exception of day 2 at 25 °C and 35 °C and day 8 at 30 °C and 40 °C (Supplementary Figure 2, *P* < 0.05). Total fumonisin levels were mostly below 1 μg/g on day 2. However, 66787 accumulated significantly more fumonisin on day 2 at 20 °C and below, suggesting that this strain started to produce fumonisins earlier than the other strains. Nevertheless, on day 2 at 25 °C, strains 66476 and 66787 accumulated comparable levels of fumonisins. On day 5 at 10 and 15 °C, 66787 accumulated significantly more fumonisins than the other strains. However, on day 5 at temperatures above 20 °C, 66476 accumulated significantly more fumonisins than the other strains. On day 8, where significant differences were detected, 66476 usually produced the highest or among the highest levels of fumonisins. Temperature also contributed to significant differences in fumonisins among strains (*P* < 0.0001). Considering all strains, fumonisin levels were highest at 25 °C, followed by 30 °C, 20 °C, 35 °C, and minimal at the low (10–15 °C) and high extreme (40 °C) temperatures (Figure 4b). However, the optimal temperature for fumonisin accumulation, represented by highest fumonisin levels, was strain dependent. 66476, 66786, ISU 180 exhibited highest mean fumonisins at 25 °C, while 66787 and 66790 had highest fumonisin means at 35 °C. Nevertheless, only 66787 and ISU 180 had significantly highest fumonisins at 25 °C in comparison to other temperatures (Tukey's HSD, *P* < 0.001), while others had a range of temperatures. No significant difference was detected between 20–25 °C for 66786, 20–30 °C for 66476 and 25–30 °C for 66790. This suggests that individual strains can have distinct optimal ranges of temperatures for fumonisin accumulation.

**Figure 5:**
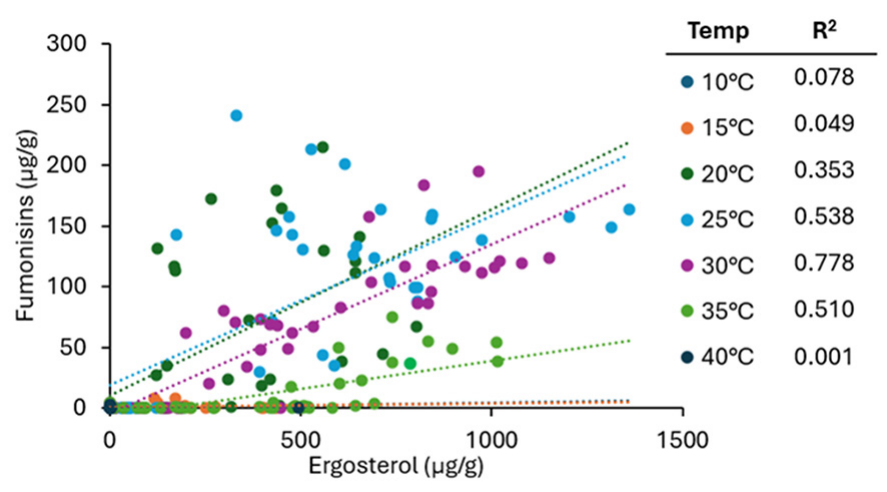
Results of regression analysis of ergosterol levels (growth) and total fumonisin content of *F. verticillioides* strains sorted by temperature. Individual temperatures are indicated by different colors.

Given the different patterns of growth and fumonisin content among strains, a regression analysis was used to evaluate the relationship between the two variables (Figure 5). In general, there was a positive relationship between the two variables, with fumonisin levels increasing as ergosterol levels increased. For all data points, the R^2^ value was 0.524. However, when evaluated by temperature, the relationship between biomass and fumonisin levels varied. The highest correlation between ergosterol and total fumonisin content occurred at 30 °C (R^2^ = 0.78; Figure 5). The markedly different R^2^ values at different temperatures emphasize that the temperature range for fumonisin production is narrower than for growth.

**Figure 6:**
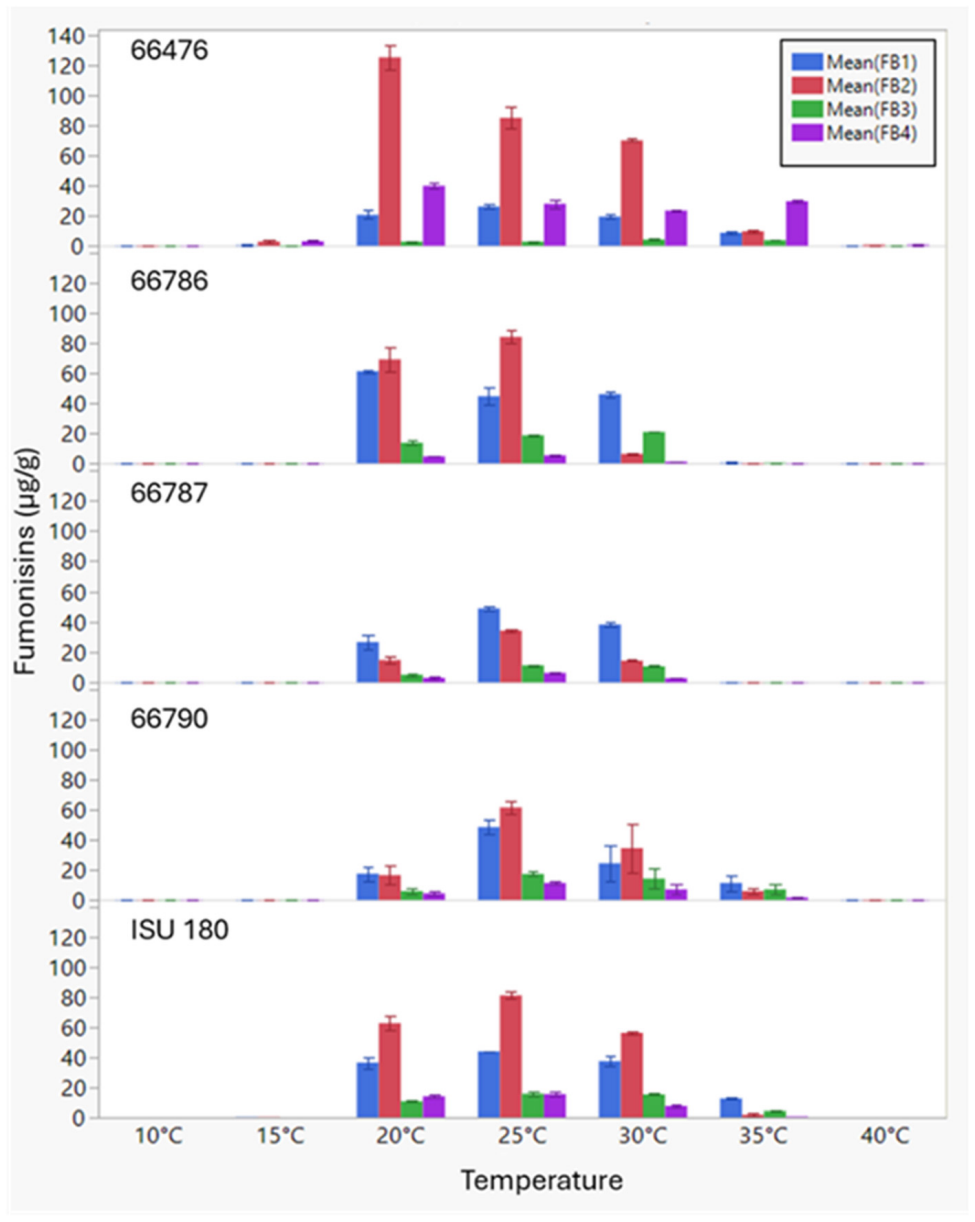
Variation in production of the B-series fumonisin analogs FB_1_-FB_4_ in five strains of *F. verticillioides* at a temperatures range of 10–40 °C. Error bars indicate standard error.

In addition to strain-specific differences in total fumonisin content, the proportions of analogs also varied by strain (Figure 6 and Supplementary Figure 3). Strain 66476, which had the highest total fumonisin content, produced higher levels of FB_2_ and FB_4_ than FB_1_ and FB_3_ compared to the other strains (Tukey HSD, *P* < 0.001). In contrast, 66786 produced the largest amounts of FB_1_ and FB_3_. 66786 produced significantly more FB_1_ than all other strains evaluated (*P* < 0.05), but the amount of FB_3_ produced by 66786 was not significantly different from ISU 180 (*P* = 0.115). ISU 180 produced relatively high amounts of all four analogs. The mean amount of FB_1_, FB_2_ and FB_4_ at 25 °C was significantly greater than other evaluated temperatures (Tukey HSD, *P* < 0.01). Only for FB3 there was no significant difference between the amount produced at 25 °C and 30 °C (*P* = 0.882). Of the 21 possible pair wise comparisons (7 temperatures and 3 time points) assessed by Tukey HSD, significant strain specific differences were detected for 15 of the FB_1_ means, 20 of the FB_2_ means, 17 of the FB_3_ means and 21 of the FB_4_ means (Supplementary Figure 3). Thus, FB_2_ and FB_4_ displayed the greatest variability. These two analogs are end-products of one branch of the fumonisin biosynthetic pathway, whereas FB_1_ and FB_3_ are end-products of another branch (Figure 8). To further visualize the strain by temperature interactions on production of fumonisin analogs in the context of these two pathway branches, the ratio of the sum of FB_1_ and FB_3_ to the sum of FB_2_ and FB_4_ (FB_1_ + FB_3_/FB_2_ + FB_4_ ratio) was calculated, and a full factorial model with fit least squares means statistical analysis was performed (Figure 6). The effects test indicated that all factors and interactions were significant contributors to differences (*P* < 0.0001), showing that the ratio of FB_1_ + FB_3_/FB_2_ + FB_4_ was time, temperature and strain dependent and that strains responded differently over time and to temperature. Interestingly, at 25 °C when overall total fumonisin levels were highest, the average ratio of FB_1_ + FB_3_/FB_2_ + FB_4_ was significantly lower than other temperatures when fumonisins were produced (Figure 4c). The highest ratio of FB_1_ + FB_3_/FB_2_ + FB_4_ was at 35 °C. The significantly higher ratio of FB_1_ + FB_3_/FB_2_ + FB_4_ at 35 °C, 30 °C, 20 °C relative to values at 25 °C, revealed a disproportionate decline in FB_2_ + FB_4_. While all fumonisin types declined at suboptimal temperatures (anything above or below 25 °C), the decline was greatest for FB_2_ and FB_4_. This decline is more apparent in strains which on average produced similar amounts of FB_1_ + FB_3_ and FB_2_ + FB_4_, such as 66786 and 66790 (Figure 6). This difference suggests that suboptimal temperatures for fumonisin production favors maintaining FB_1_ and FB_3_ biosynthetic pathways over FB_2_ and FB_4_.

**Figure 7:**
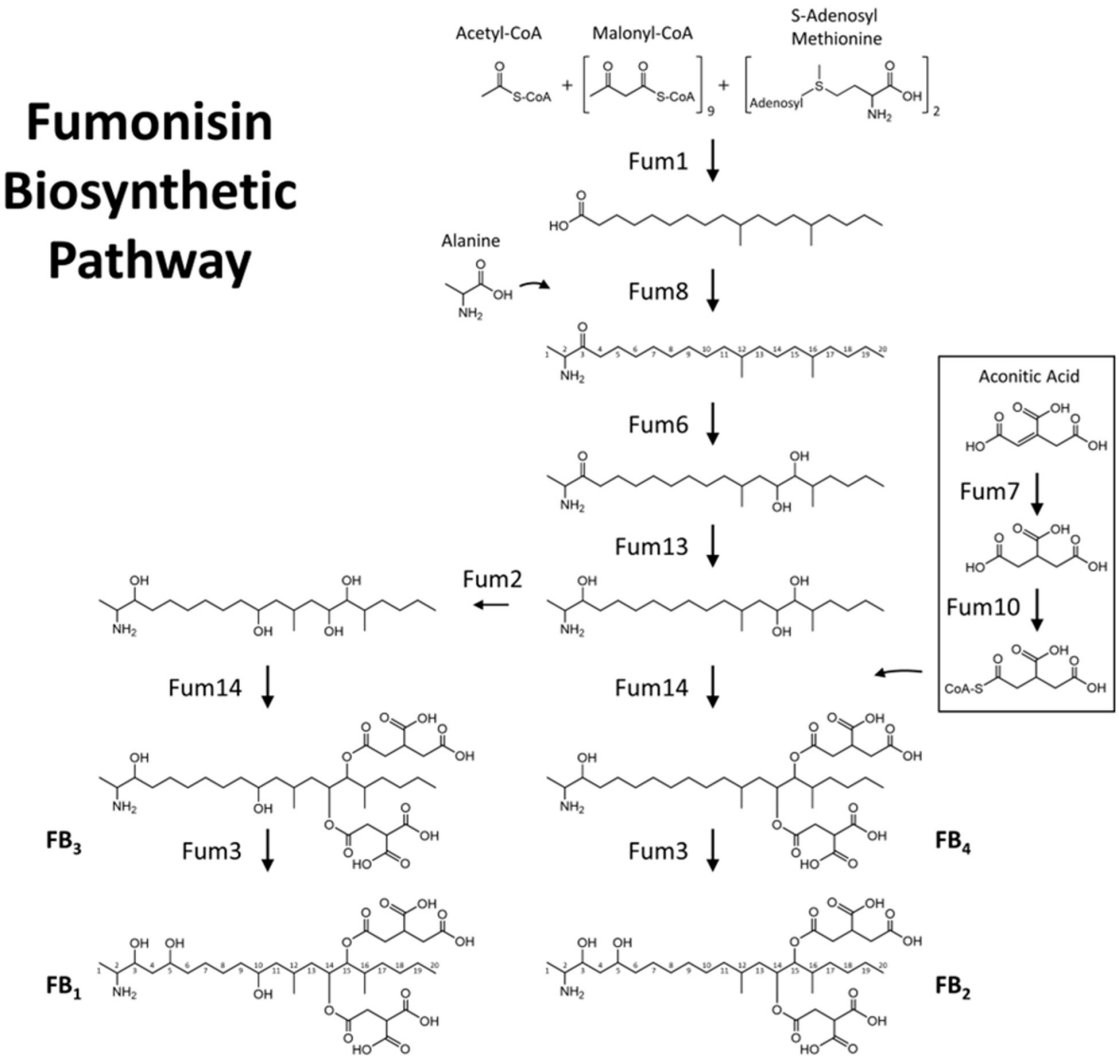
Proposed biosynthetic pathway for the B-series fumonisin analogs FB_1_-FB_4_ in *F. verticillioides*.

**Figure 8:**
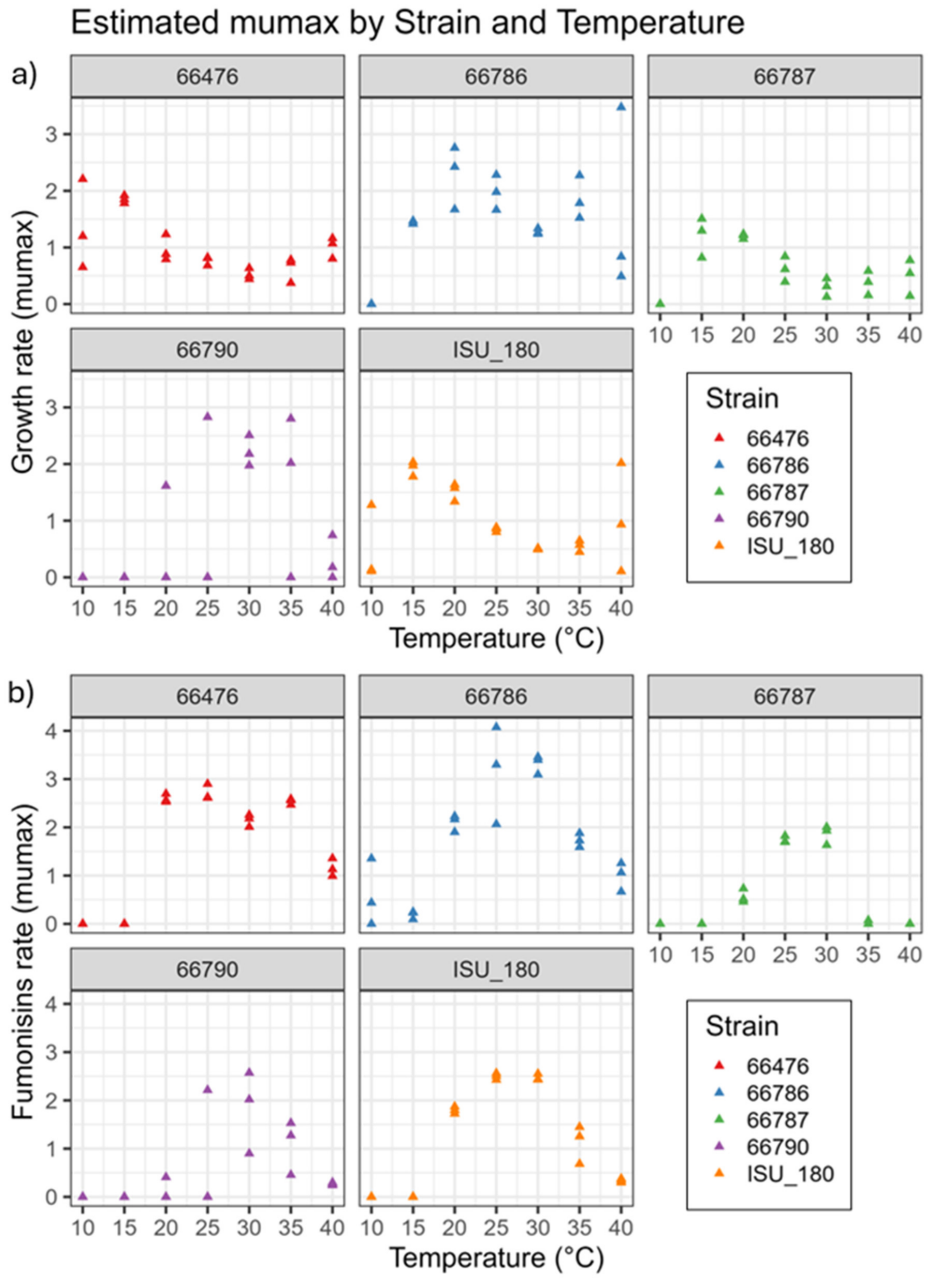
Baranyi fitted **(a)** growth and **(b)** toxin production rate (mumax) at 10, 15, 20, 25, 30, 35 and 40 °C. Strains evaluated were NRRL 66786 (Blue color), 66476 (Red color), 66787 (Green color), 66790 (Purple color), and ISU 180 (Orange color). Y-axis represents fitted rate (mumax) and X-axis represents temperature.

For the development of risk indexes, the maximum rate (mumax) of growth and fumonisin content was estimated by using the Baranyi growth model (Figure 8). Growth rates varied by strain, particularly at the two highest temperatures evaluated (35 and 40 °C) where the mumax of all the replicates was above zero for strains 66476, 66786, and 66787 (Figure 8a). In the case of strains 66790 and ISU 180, one out of three replicates had a mumax equal to zero, while the others were above that value (Figure 8a). Fumonisin mumax had several zero values at the lowest temperatures (10 and 15 °C) for strains 66476, 66787, 66790 and ISU 180 while only strain 66787 had zero rate values at the highest temperature (40 °C) (Figure 8b).

**Figure 9:**
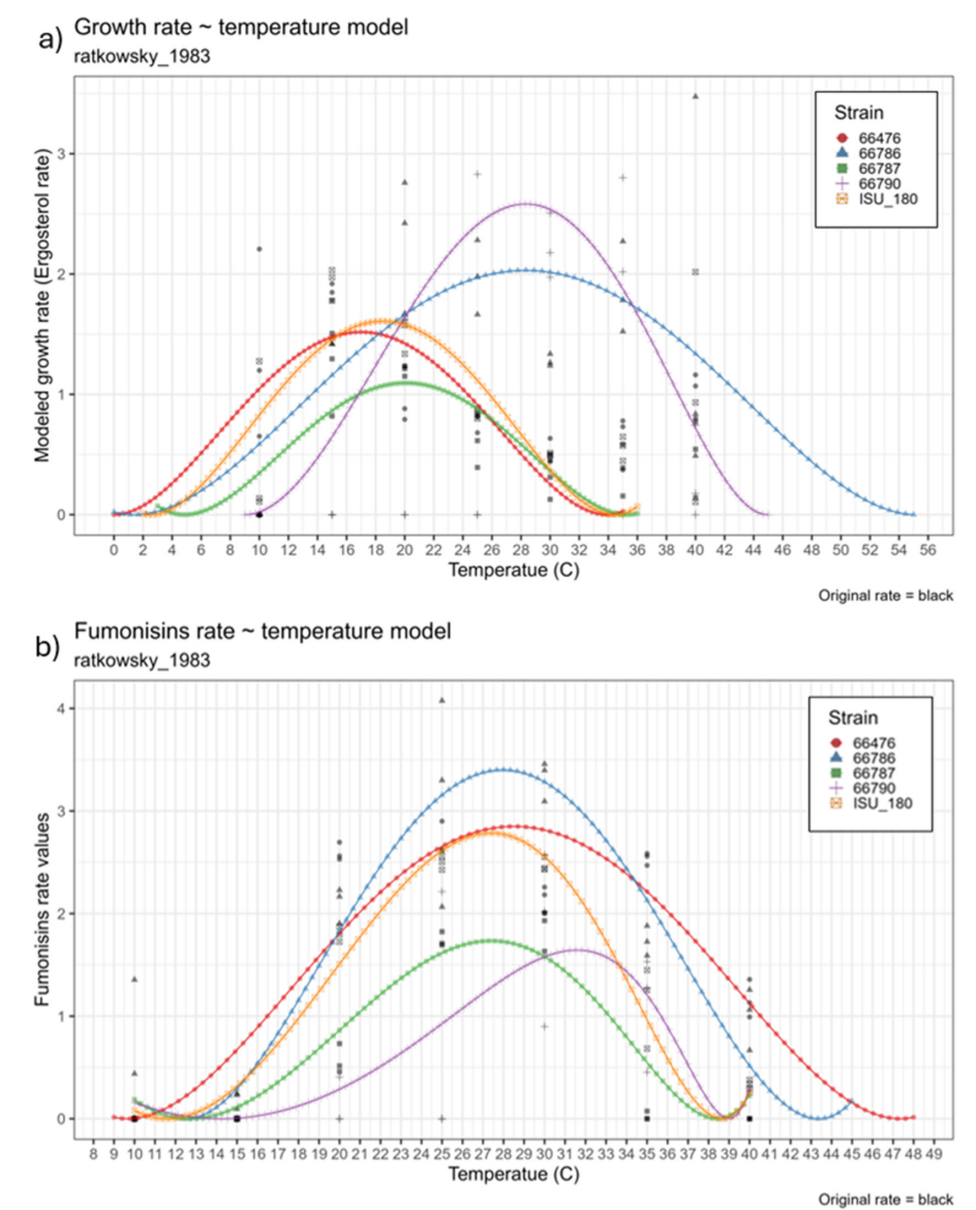
Ratkowsky fit models for growth **(a)** and total fumonisin content **(b)**. Ratkowsky model-derived values for minimum and maximum temperatures at which growth or fumonisin production occurred and temperatures at which the maximum rate of growth or fumonisin production occurred are provided in Tables 1–9. Strains evaluated were NRRL 66786 (Blue color), 66476 (Red color), 66787 (Green color), 66790 (Purple color), and ISU 180 (Orange color).

Finally, the Baranyi fitted rates were used to develop the Ratkowsky model equation for growth and fumonisin production for each strain. This analysis revealed that the fitted minimum temperature (tmin) at which growth occurred was variable among the strains (Table 1), from 0 °C for strain 66476 to 9 °C for strain 66790. Likewise, there was high variation in the maximum temperature (tmax) at which growth occurred, from 34 °C for strain 66476 to 55 °C for strain 66786 (Table 1). Similarly, the minimum and maximum temperatures (tmin and tmax) at which fumonisins were produced was broad. The tmin for fumonisin production ranged from 10 to 14 C, and the tmax for fumonisin production ranged from 38 to 47 °C (Table 2). Visualization of the Ratkowsky fit curves for both fungal growth and fumonisin production rates illustrate the breadth of strain-specific growth and fumonisin production range differences. Strain 66786 had the widest growth range temperature distribution, with maximum growth rate of 2.03 at 28 °C (Figure 9a and Table 3), however the highest maximum growth rate, 2.85, was achieved by 66790 at 28 °C (Figure 9a and Table 3). The other strains had lower maximum growth rates that occurred at lower temperatures (between 16 and 20 °C). In contrast, the fumonisin production rates had a narrower range as illustrated by the Ratkowsky fit curves (Figure 9b and Table 3). Strain 66786 had the highest fumonisins production rate, 3.4 at 28 °C (Figure 9b and Table 3), while strain 66476 had the widest temperature range of fumonisin production but a lower maximum production rate.

**Table 1:** Ratkowsky equation coefficients fitted for fungal growth rate.

**Coefficient**	**Strain_66476**	**Strain_66786**	**Strain_66787**	**Strain_66790**	**Strain_ISU_180**
tmin	0.00 °C	1.39 °C	4.84 °C	9.01 °C	2.51 °C
tmax	33.89 °C	55.21 °C	35.32 °C	44.95 °C	34.44 °C
a	7.27	26.11	70.19	0.33	63.82
b	5.933155e-04	7.545982e-05	6.421785e-05	0.01766	7.804855e-05

Strains evaluated were NRRL 66786, 66476, 66787, 66790, and ISU 180.

**Table 2:** Ratkowsky equation coefficients fitted for fumonisins production rate.

**Coefficient**	**Strain_66476**	**Strain_66786**	**Strain_66787**	**Strain_66790**	**Strain_ISU_180**
tmin	9.67 °C	12.54 °C	12.71 °C	14.21 °C	11.57 °C
tmax	47.34 °C	43.37 °C	38.41 °C	39.01 °C	38.66 °C
a	40.52	87.07	0.21	0.09	0.22
b	1.175306e-04	8.922485e-05	0.0500297	0.20394422	0.05707556

Strains evaluated were NRRL 66786, 66476, 66787, 66790, and ISU 180.

**Table 3:** Ratkowsky equation-derived temperatures and rates for growth and total fumonisin production in five *Fusarium verticillioides* strains.

**Strain**	**Growth**		**Total fumonisin content**	
	**Temperature (**°**C)**	**Maximum rate**	**Temperature (**°**C)**	**Maximum rate**
66476	16.96	1.52	28.69	2.85
66786	28.33	2.03	28.00	3.40
66787	20.00	1.09	27.27	1.73
66790	28.27	2.58	31.51	1.64
ISU_180	18.48	1.61	27.27	2.78
Mean	21.92	1.82	28.55	2.48
Standard deviation	4.91	0.67	1.76	0.77

## 4 Discussion

This study is the first evaluation of how genetic diversity of *F. verticillioides* impacts development of a mathematically engineered feature, fumonisin risk index, that links fungal growth and mycotoxin production rates with temperature in the environment (Ratkowsky and Reddy, 2017). The findings reveal the importance of understanding genetic diversity of the fungus and how this diversity influences growth, as an indicator of inoculum potential, and fumonisin production. Significant strain-specific differences were identified, providing evidence that temperature ranges and optima as well as magnitude of growth and fumonisin production varied with genetic background of *F. verticillioides* strains.

In an early study, Alberts et al. observed that *F. verticillioides* strain MRC 826 had maximum growth and FB1 production at 20 and 25 °C (Alberts et al., 1990). Marin et al. identified 30 °C as the optimal temperature for FB_1_ production by *F. verticillioides* (Marin et al., 1999). Later, Samapundo et al. evaluated *F. verticillioides* strain 25N growth at variable a_w_ and temperatures (15–30 °C) and showed that 0.975 a_w_ and 25 °C resulted in optimal colony growth (Samapundo et al., 2005). Our observations herein largely are in agreement with these previous reports (Samapundo et al., 2005; Alberts et al., 1990; Marin et al., 1999), but include a larger number of genetically distinct strains thereby allowing for greater confidence in models that are more inclusive of the potential *F. verticillioides* diversity in field populations.

Furthermore, the findings from our study provide valuable insights into the genetic diversity and strain-specific behavior of *F. verticillioides*, particularly concerning growth and fumonisin production under varying temperatures. The phylogenetic analysis revealed two distinct clades of *F. verticillioides*, suggesting significant genetic variation within the species. This diversity highlights the importance of selecting multiple strains for evaluating fumonisin risk, as it enables a more comprehensive understanding of how temperature influences growth and mycotoxin production in the species. The analysis of growth and total fumonisin content emphasized the complex interactions between strain, temperature, and time.

The Ratkowsky fitted models showed that strain influenced the variability of the distribution and magnitude of tmin and tmax, as well as the magnitude of the modeled maximum rate of growth and fumonisin production (Figure 9). This would suggest that strain diversity had a strong effect on temperature range for growth, and an effect on the magnitude of growth and fumonisin production rate. The larger standard deviation for the strains' maximum growth and fumonisin production rates, and the temperatures that correspond to those rates further quantify the strain specific effect on variability of outcomes (Table 3). The differences in tmin, tmax, maximum growth, and fumonisin production rates could be associated with the strains genetic background and/or geographic origin. Some results suggest the possibility of region-specific risk factors in addition to those resulting from the observed genomic diversity. The Ratkowsky curves (Figure 9) and temperatures for maximum rate of growth and fumonisin production (Table 3) were similar for the two strains (ISU 180 and 66476) from the Midwestern United States Corn Belt, even though these strains were relatively distantly related (Figure 1). Likewise, temperatures for maximum rate of growth were similar for the two strains (66786 and 66790) from Nepal (Table 3). However, the Ratkowsky curve and temperature for maximum rate of fumonisin production differed substantially for the two Nepal strains (Figure 9). Therefore, the strain-dependent nature of optimal and range of conditions for growth and fumonisin production suggests that prediction models for fumonisin contamination could be more accurate if they include information about *F. verticillioides* diversity within the fields. However, this would require a more comprehensive analysis of regional specific differences in *F. verticillioides* populations. Analysis of growth and fumonisin production of additional strains of *F. verticillioides* from different regions could provide evidence for whether there are indeed region-specific factors that impact fumonisin risk. Further, more thorough analyses of genome sequence data, e.g., whole-genome single nucleotide polymorphism analysis, could identify sequence variation that is correlated with the putative region-specific risk factors.

Additionally, the Ratkowsky fitted equation and constants showed that on average the maximum rate for growth occurred at approximately 7 °C lower than the maximum rate for fumonisin production except for strain 66786 where the two maximum production rates happened at the same temperature, ~28 °C (Table 3). This has key repercussions on the in-field usage of fumonisin risk index. Thus, strains with wide growth or production curves may be capable of growing across a broad temperature range. However, if their maximum growth or toxin production rates are low, such strains may still be competitively disadvantaged in the presence of strains with higher rates. Thus, thermal breadth and competitive ability are distinct biological attributes.

Strain specific temperature adaptations are also worth noting, for example, Strain 66787 demonstrated the ability to produce fumonisins earlier than other strains at temperatures below 20 °C (Supplementary Figure 2). This early production could pose a risk, especially if conditions remain favorable for growth. Strain 66476 displayed substantial fumonisin accumulation at higher temperatures (Figure 2 and Supplementary Figure 2). Also, the optimal temperature for growth was 25–35 °C, with different strains exhibiting different optima. According to LS means results, the growth optimum for strains ISU 180, 66787, and 66786 was 25 °C, whereas the optimum for strains 66476 and 66790 was 30 °C (Figure 3). The high levels of growth of strain 66476 at 35 °C suggests thermotolerance that could give it a competitive advantage in warmer regions. Together, these data emphasize the need for monitoring and characterizing pathogen populations and diversity. Precision agricultural management strategies that take into account pathogen population characteristics and diversity in fields will likely be most effective. Thus, a fumonisin risk index incorporating strain specific temperature responses could support precision agriculture by guiding hybrid selection in regions where thermotolerant strains predominate, informing optimal timing of insect management to reduce wounding mediated infection during high-risk temperature windows, and enabling targeted fungicide or biocontrol deployment when environmental conditions align with strain specific risk thresholds.

Furthermore, the delayed growth of strains 66786 and 66790, as evident by lower levels of ergosterol at day 2 relative to other strains, was followed by a notable increase in growth on day 5 at 25, 30, and/or 35 °C (Supplementary Figure 1). This suggests the potential for individual strains in a field population to have slower metabolic activation or to require longer adaptation phases at certain temperatures, but such lags could be compensated for later. This behavior emphasizes the importance of considering temporal dynamics in fungal growth when assessing fumonisin risk. Based on these temporal dynamics, it may be that unique formulas that assess risk of inoculum formation and fumonisin accumulation at the different timepoints within the growing season and crop phenological states, would provide higher predictive power to models. Furthermore, this strategy could provide farmers with two distinct opportunities to implement mitigation strategies and prevent inoculum as well as fumonisin accumulation at the more appropriate corresponding crop developmental stage.

Fumonisin production is most often reported as total production, or only one selected analog, usually FB_1_. Herein we report on levels of all four B analogs (FB_1_-_4_) and their different trends for production at different temperatures by genetically diverse strains. In *F. verticillioides*, a positive correlation has been demonstrated between fumonisin production and expression of the gene that encodes a polyketide synthase that catalyzes formation of the carbon chain that forms most of the backbone structure of fumonisins (Lopez-Errasquin et al., 2007). The biosynthetic process for adding the hydroxyl groups necessary for formation of FB_1_ and FB_3_ is catalyzed by the Fum2 enzyme (Proctor et al., 2006). Isolates of *F. verticillioides* lacking a functioning Fum2 have been observed to only produce FB_2_ and FB_4_, with a total lack of FB_1_ and FB_3_ production. Our results demonstrate that there are strain specific differences in the proportion of fumonisins with the C_10_ hydroxylation (FB_1_ and FB_3_) and that these proportions are affected by temperature.

Previously, differential expression of gene cluster genes in aflatoxin producing isolates of *Aspergillus flavus* has been shown to result in varying ratios of AFB_1_ vs. AFG_1_. The differential gene expression in *A. flavus* isolates was observed to result from environmental factors such as temperature and water activity (Schmidt-Heydt et al., 2010). Here, we see a general increase in relative levels of FB_1_ + FB_3_ to FB_2_ + FB_4_ at higher temperatures. Preference for FB_1_ + FB_3_ over FB_2_ + FB_4_ may reflect results of a temperature influenced competition between Fum2 vs. Fum14 enzymes in the fumonisin pathway. An increased relative level of FB_1_ + FB_3_ to FB_2_ + FB_4_ at higher indicates a possible higher tolerance toward elevated temperature for Fum2, as compared to Fum14.

Nevertheless, suboptimal temperatures for fumonisin production appear to favor maintaining C_10_ hydroxylation resulting in higher proportions of FB_1_ and FB_3_ over FB_2_ and FB_4_ (Figure 6 and Supplementary Figure 3). Since FB_1_ is the most toxic of the analogs (Henry and Wyatt, 2001; Gao et al., 2023; Efsa Panel on Contaminants in the Food Chain et al., 2018; Chen et al., 2021), the shift in proportions seems to favor toxicity. This adds an additional layer of complexity that could have repercussions on total fumonisin toxicity based on the analog proportions.

## 5 Conclusions

This work demonstrates that *F. verticillioides* growth and fumonisin production are governed by a complex interplay of strain genetics, temperature, and time, with distinct optima for biomass accumulation and toxin synthesis. There are specific conditions where the fungus thrives and produces toxins, but these conditions vary significantly between different strains. Notably, the range of temperatures that allows for fumonisin production is narrower than what the fungus needs for growth. This finding highlights why a one-size-fits-all approach to risk assessment might not be effective. Interestingly, there is a positive relationship between the biomass of the fungus and the levels of toxins, but this is also dependent on temperature. This suggests that monitoring efforts should consider both the environmental factors at play and the fungal diversity within the fields. By quantifying growth and toxin production rates and revealing shifts in analog ratios under suboptimal conditions, this study provides critical parameters for refining predictive models and early warning systems. These models will be crucial for reducing fumonisin contamination in maize, thereby protecting US farmers profitability and ensuring safety and health of consumers.

## Data Availability

The datasets presented in this study can be found in online repositories. The names of the repository/repositories and accession number(s) can be found in the article/Supplementary material.

## References

[B1] Alberts J. F. Gelderblom W. C. Thiel P. G. Marasas W. F. O. Van Schalkwyk D. J. Behrend Y. (1990). Effects of temperature and incubation period on production of fumonisin b1 by Fusarium moniliforme. Appl. Environ. Microbiol. 56, 1729–1733. doi: 10.1128/aem.56.6.1729-1733.1990 2383011 PMC184501

[B2] Baranyi J. Roberts T. A. (1995). Mathematics of predictive food microbiology. Int. J. Food Microbiol. 26, 199–218. doi: 10.1016/0168-1605(94)00121-L 7577358

[B3] Blacutt A. A. Gold K. A. Voss K. A. Gao M. Glenn A. E. (2018). Fusarium verticillioides: advancements in understanding the toxicity, virulence, and niche adaptations of a model mycotoxigenic pathogen of maize. Phytopathology 108, 312–326. doi: 10.1094/PHYTO-06-17-0203-RVW 28971734

[B4] Branstad-Spates E. Castano-Duque L. Mosher G. Hurburgh C. Rajasekaran K. Owens P. . (2024). Predicting fumonisins in iowa corn: gradient boosting machine learning. Cereal Chem. 101, 1261–1272. doi: 10.1002/cche.10824 PMC10502509 37720139

[B5] Castano-Duque L. Avila A. Mack B. M. Winzeler H. E. Blackstock J. M. Lebar M. D. . (2025). Prediction of aflatoxin contamination outbreaks in texas corn using mechanistic and machine learning models. Front. Microbiol. 16:1528997. doi: 10.3389/fmicb.2025.1528997 40109977 PMC11919900

[B6] Castano-Duque L. Vaughan M. Lindsay J. Barnett K. Rajasekaran K. (2022). Gradient boosting and bayesian network machine learning models predict aflatoxin and fumonisin contamination of maize in illinois - first usa case study. Front. Microbiol. 13:1039947. doi: 10.3389/fmicb.2022.1039947 36439814 PMC9684211

[B7] Castano-Duque L. Winzeler E. Blackstock J. M. Liu C. Vergopolan N. Focker M. . (2023). Dynamic geospatial modeling of mycotoxin contamination of corn in illinois: unveiling critical factors and predictive insights with machine learning. Front. Microbiol. 14:1283127. doi: 10.3389/fmicb.2023.1283127 38029202 PMC10646420

[B8] Chen J. Wen J. Tang Y. Shi J. Mu G. Yan R. . (2021). Research progress on fumonisin b1 contamination and toxicity: a review. Molecules 26:5238. doi: 10.3390/molecules26175238 34500671 PMC8434385

[B9] de la Campa R. Hooker D. C. Miller J. D. Schaafsma A. W. Hammond B. G. (2005). Modeling effects of environment, insect damage, and bt genotypes on fumonisin accumulation in maize in argentina and the philippines. Mycopathologia 159, 539–552. doi: 10.1007/s11046-005-2150-3 15983741

[B10] Degradi L. Tava V. Esposto M. C. Prigitano A. Bulgari D. Kunova A. . (2024). Genomic insights into Fusarium verticillioides diversity: the genome of two clinical isolates and their demethylase inhibitor fungicides susceptibility. Pathogens 13:1062. doi: 10.3390/pathogens13121062 39770322 PMC11728828

[B11] Dey A. Bokka V. Sen S. (2020). Dependence of bacterial growth rate on dynamic temperature changes. IET Syst. Biol. 14, 68–74. doi: 10.1049/iet-syb.2018.5125 32196465 PMC8687403

[B12] Dowd P. F. Barnett J. Bartelt R. J. Beck J. J. Berhow M. A. Duvick J. P. . (2004). Insect management for reduction of mycotoxins in midwest corn. fy-2002 report. Mycopathologia 157:474.

[B13] Efsa Panel on Contaminants in the Food Chain Knutsen, H. K. Alexander J. Barregard L. Bignami M. Bruschweiler B. . (2018). Risks for animal health related to the presence of fumonisins, their modified forms and hidden forms in feed. EFSA J. 16:e05242. doi: 10.2903/j.efsa.2018.5242 32625894 PMC7009563

[B14] Gao Z. Luo K. Zhu Q. Peng J. Liu C. Wang X. . (2023). The natural occurrence, toxicity mechanisms and management strategies of fumonisin b1:a review. Environ. Pollut. 320:121065. doi: 10.1016/j.envpol.2023.121065 36639041

[B15] Hall B. G. Acar H. Nandipati A. Barlow M. (2014). Growth rates made easy. Mol. Biol. Evol,. 31, 232–238. doi: 10.1093/molbev/mst187 24170494

[B16] Henderson C. M. Lozada-Contreras M. Naravane Y. Longo M. L. Block D. E. (2011). Analysis of major phospholipid species and ergosterol in fermenting industrial yeast strains using atmospheric pressure ionization ion-trap mass spectrometry. J. Agric. Food Chem. 59, 12761–12770. doi: 10.1021/jf203203h 21995817

[B17] Henry M. H. Wyatt R. D. (2001). The toxicity of fumonisin b1, b2, and b3, individually and in combination, in chicken embryos. Poult. Sci. 80, 401–407. doi: 10.1093/ps/80.4.401 11297276

[B18] Hudson O. Meinecke C. D. Brawner J. T. (2024). Comparative genomics of Fusarium species causing Fusarium ear rot of maize. PLoS ONE 19:e0306144. doi: 10.1371/journal.pone.0306144 39423180 PMC11488721

[B19] Jacquat A. G. Podio N. S. Canizares M. C. Velez P. A. Theumer M. G. Areco V. A. . (2025). The growth, pathogenesis, and secondary metabolism of Fusarium verticillioides are epigenetically modulated by putative heterochromatin protein 1 (fvhp1). J. Fungi 11:424. doi: 10.3390/jof11060424 40558936 PMC12194461

[B20] Kumar S. Stecher G. Tamura K. (2016). Mega7: molecular evolutionary genetics analysis version 7.0 for bigger datasets. Mol. Biol. Evol. 33, 1870–1874. doi: 10.1093/molbev/msw054 27004904 PMC8210823

[B21] Lanubile A. Bellin D. Ottaviani L. Jaberi M. Marocco A. Mule G. . (2025). Effect of fumonisin b on transcriptional profiles and biochemical signatures in resistant and susceptible maize shoots. J. Plant Growth Regul. 44, 2514–2528. doi: 10.1007/s00344-024-11564-9

[B22] Lopez-Errasquin E. Vazquez C. Jimenez M. Gonzalez-Jaen M. T. (2007). Real-time rt-pcr assay to quantify the expression of fum1 and fum19 genes from the fumonisin-producing Fusarium verticillioides. J. Microbiol. Methods 68, 312–317. doi: 10.1016/j.mimet.2006.09.007 17055092

[B23] Marasas W. F. O. (2001). Discovery and occurrence of the fumonisins: a historical perspective. Environ. Health Perspect. 109, 239–243. doi: 10.1289/ehp.01109s2239 11359691 PMC1240671

[B24] Marin S. Magan N. Belli N. Ramos A. J. Canela R. Sanchis V. (1999). Two-dimensional profiles of fumonisin b1 production by Fusarium moniliforme and Fusarium proliferatum in relation to environmental factors and potential for modelling toxin formation in maize grain. Int. J. Food Microbiol. 51, 159–167. doi: 10.1016/S0168-1605(99)00115-4 10574091

[B25] Miller J. D. (2001). Factors that affect the occurrence of fumonisin. Environ. Health Perspect. 109, 321–324. doi: 10.1289/ehp.01109s2321 11359702 PMC1240682

[B26] Missmer S. A. Suarez L. Felkner M. Wang E. Merrill A. H. Jr. Rothman K. J. . (2006). Exposure to fumonisins and the occurrence of neural tube defects along the texas-mexico border. Environ. Health Perspect. 114, 237–241. doi: 10.1289/ehp.8221 16451860 PMC1367837

[B27] Munkvold G. P. (2003). Epidemiology of Fusarium diseases and their mycotoxins in maize ears. Eur. J. Plant Pathol. 109, 705–713. doi: 10.1023/A:1026078324268

[B28] Munkvold G. P. Desjardins A. E. (1997). Fumonisins in maize: can we reduce their occurrence? Plant Dis. 81, 556–565. doi: 10.1094/PDIS.1997.81.6.556 30861834

[B29] Munkvold G. P. Hellmich R. L. Rice L. G. (1999). Comparison of fumonisin concentrations in kernels of transgenic bt maize hybrids and nontransgenic hybrids. Plant Dis. 83, 130–138. doi: 10.1094/PDIS.1999.83.2.130 30849794

[B30] Munkvold G. P. Hellmich R. L. Showers W. B. (1997a). Reduced fusarium ear rot and symptomless infection in kernels of maize genetically engineered for european corn borer resistance. Phytopathology 87, 1071–1077. doi: 10.1094/PHYTO.1997.87.10.1071 18945043

[B31] Munkvold G. P. McGee D. C. Carlton W. M. (1997b). Importance of different pathways for maize kernel infection by Fusarium moniliforme. Phytopathology 87, 209–217. doi: 10.1094/PHYTO.1997.87.2.209 18945144

[B32] Nguyen L. T. Schmidt H. A. von Haeseler A. Minh B. Q. (2015). Iq-tree: a fast and effective stochastic algorithm for estimating maximum-likelihood phylogenies. Mol. Biol. Evol. 32, 268–274. doi: 10.1093/molbev/msu300 25371430 PMC4271533

[B33] Niehaus E. M. Kim H. K. Munsterkotter M. Janevska S. Arndt B. Kalinina S. A. . (2017). Comparative genomics of geographically distant Fusarium fujikuroi isolates revealed two distinct pathotypes correlating with secondary metabolite profiles. PLoS Pathog. 13:e1006670. doi: 10.1371/journal.ppat.1006670 29073267 PMC5675463

[B34] Opoku J. Kleczewski N. M. Hamby K. A. Herbert D. A. Malone S. Mehl H. L. (2019). Relationship between invasive brown marmorated stink bug (Halyomorpha halys) and fumonisin contamination of field corn in the mid-atlantic U.S. Plant Dis. 103, 1189–1195. doi: 10.1094/PDIS-06-18-1115-RE 30964416

[B35] Plattner R. D. (1999). HPLC/MS analysis of fusarium mycotoxins, fumonisins and deoxynivalenol. Nat. Toxins 7, 365–370. 11122531

[B36] Proctor R. H. Plattner R. D. Desjardins A. E. Busman M. Butchko R. A. (2006). Fumonisin production in the maize pathogen Fusarium verticillioides: genetic basis of naturally occurring chemical variation. J. Agric. Food Chem. 54, 2424–2430. doi: 10.1021/jf0527706 16536629

[B37] Proctor R. H. Van Hove F. Susca G. Stea G. Busman M. van der Lee T. . (2013). Birth, death and horizontal transfer of the fumonisin biosynthetic gene cluster during the evolutionary diversification of Fusarium. Mol. Microbiol. 90, 290–306. doi: 10.1111/mmi.12362 23937442

[B38] R Core Team (2017). R: A Language and Environment for Statistical Computing. Vienna: R Foundation for Statistical Computing.

[B39] Ratkowsky D. A. Lowry R. K. McMeekin T. A. Stokes A. N. Chandler R. E. (1983). Model for bacterial culture growth rate throughout the entire biokinetic temperature range. J. Bacteriol. 154, 1222–1226. doi: 10.1128/jb.154.3.1222-1226.1983 6853443 PMC217594

[B40] Ratkowsky D. A. Reddy G. V. P. (2017). Empirical model with excellent statistical properties for describing temperature-dependent developmental rates of insects and mites. Ann. Entomol. Soc. Am. 110, 302–309. doi: 10.1093/aesa/saw098

[B41] Rheeder J. P. Marasas W. F. O. Vismer H. F. (2002). Production of fumonisin analogs by Fusarium species. Appl. Environ. Microbiol. 68, 2101–2105. doi: 10.1128/AEM.68.5.2101-2105.2002 11976077 PMC127586

[B42] Samapundo S. Devliehgere F. De Meulenaer B. Debevere J. (2005). Effect of water activity and temperature on growth and the relationship between fumonisin production and the radial growth of Fusarium verticillioides and Fusarium proliferatum on corn. J. Food Prot. 68, 1054–1059. doi: 10.4315/0362-028X-68.5.1054 15895741

[B43] Schmidt-Heydt M. Rufer C. E. Abdel-Hadi A. Magan N. Geisen R. (2010). The production of aflatoxin b1 or g 1 by Aspergillus parasiticus at various combinations of temperature and water activity is related to the ratio of afls to aflr expression. Mycotoxin Res. 26, 241–246. doi: 10.1007/s12550-010-0062-7 23605486

[B44] Shi P.-J. Fan M.-L. Ratkowsky D. A. Huang J.-G. Wu H.-I. Chen L. . (2017). Comparison of two ontogenetic growth equations for animals and plants. Ecol. Modell. 349, 1–10. doi: 10.1016/j.ecolmodel.2017.01.012

[B45] Stafstrom W. Ngure F. Mshanga J. Wells H. Nelson R. J. Mischler J. (2025). Modeling maize aflatoxins and fumonisins in a tanzanian smallholder system: accounting for diverse risk factors improves mycotoxin models. PLoS ONE 20:e0316457. doi: 10.1371/journal.pone.0316457 39804920 PMC11729969

[B46] Vaidya G. Lohman D. J. Meier R. (2011). Sequencematrix: concatenation software for the fast assembly of multi-gene datasets with character set and codon information. Cladistics 27, 171–180. doi: 10.1111/j.1096-0031.2010.00329.x 34875773

[B47] Voss K. A. Smith G. W. Haschek W. M. (2007). Fumonisins: toxicokinetics, mechanism of action and toxicity. Anim. Feed Sci. Technol. 137, 299–325. doi: 10.1016/j.anifeedsci.2007.06.007

[B48] Waes J. G. Starr L. Maddox J. Aleman F. Voss K. A. Wilberding J. . (2005). Maternal fumonisin exposure and risk for neural tube defects: mechanisms in an in vivo mouse model. Birth Defects Res. A 73, 487–497. doi: 10.1002/bdra.20148 15959874

[B49] Wang E. Norred W. P. Bacon C. W. Riley R. T. Merrill A. H. (1991). Inhibition of sphingolipid biosynthesis by fumonisins. Implications for diseases associated with Fusarium moniliforme. J. Biol. Chem. 266, 14486–14490. doi: 10.1016/S0021-9258(18)98712-0 1860857

[B50] Wu F. (2007). Measuring the economic impacts of Fusarium toxins in animal feeds. Anim. Feed Sci. Technol. 137, 363–374. doi: 10.1016/j.anifeedsci.2007.06.010

[B51] Yates I. E. Arnold J. W. Hinton D. M. Basinger W. Walcott R. R. (2003). Fusarium verticillioides induction of maize seed rot and its control. Can. J. Bot. 81, 422–428. doi: 10.1139/b03-034

[B52] Zwietering M. H. de Koos J. T. Hasenack B. E. de Witt J. C. van't Riet K. (1991). Modeling of bacterial growth as a function of temperature. Appl. Environ. Microbiol. 57, 1094–1101. doi: 10.1128/aem.57.4.1094-1101.1991 2059034 PMC182851

