# Supplementary material for: Fusarium verticillioides genetics contribute to variability in fumonisin risk in maize
Source: Front Microbiol. 2026 Mar 3;17:1713439. doi: 10.3389/fmicb.2026.1713439 (PMC12992276; doi:10.3389/fmicb.2026.1713439)
Supplement: Supplementary file 1 [file Supplementary_file_1.docx]

Supplementary Material

# Supplementary Data

Supplementary Material should be uploaded separately on submission. Please include any supplementary data, figures and/or tables.

Supplementary material is not typeset so please ensure that all information is clearly presented, the appropriate caption is included in the file and not in the manuscript, and that the style conforms to the rest of the article.

# Supplementary Figures and Tables

For more information on Supplementary Material and for details on the different file types accepted, please see [here](https://www.frontiersin.org/guidelines/author-guidelines#supplementary-material).

## Supplementary Figures


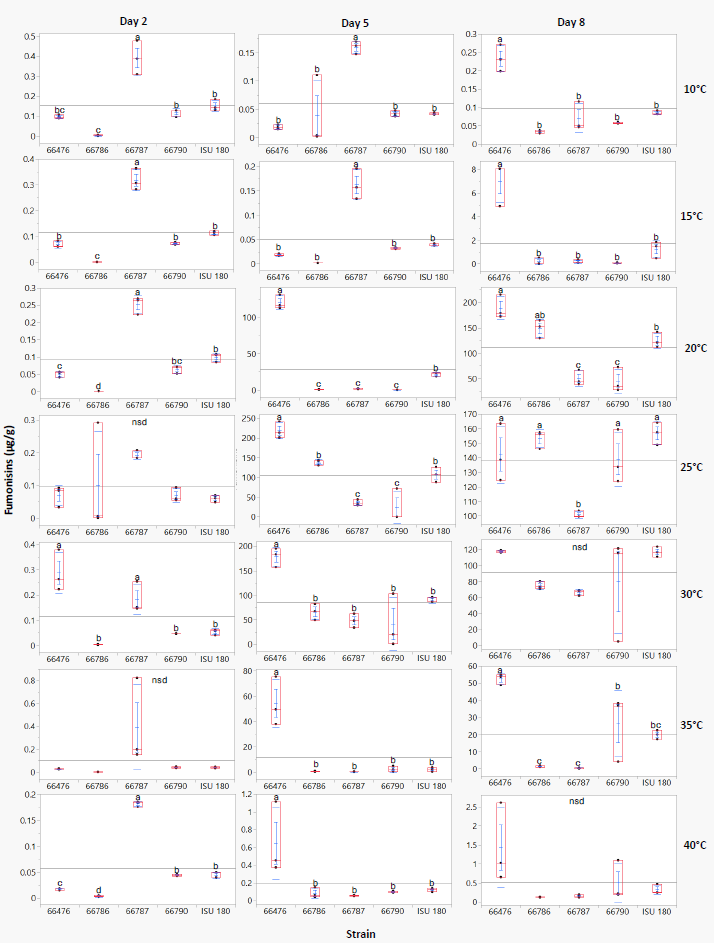


**Supplementary Figure 1.** Box plots comparing ergosterol (µg/g) levels by temperature and day for individual *F. verticillioides* strains. Ergosterol levels were estimated by LC-MS/MS. Horizontal line spanning all groups represents the mean response of all observations depicted within the panel. Red boxes represent quantiles, and blue lines represent means and standard deviations. Means not sharing the same letters above the plots are significantly different as determined by Tukey’s honestly significant difference (HSD) tests (P<0.05). No significant difference between strain biomass represented by ergosterol means are indicated as “nds” (P>0.05). Independent tests were performed for individual temperatures and days.

.

## Supplementary Figures


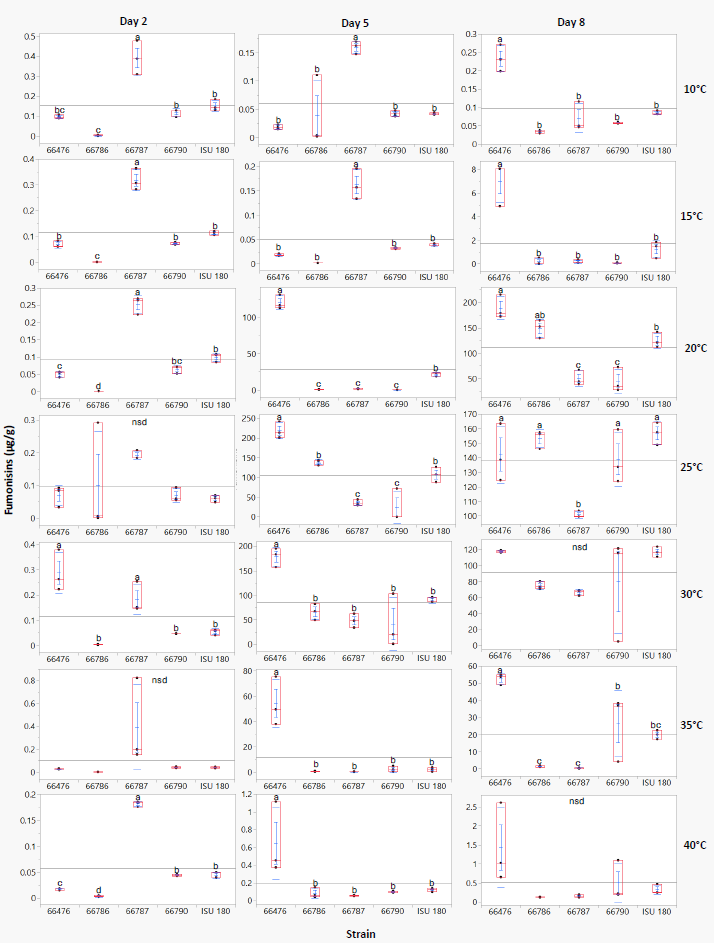


**Supplementary Figure 2.** Box plots comparing total fumonisin (µg/g) levels by temperature and day for individual *F. verticillioides* strains. Total fumonisin levels were calculated by the sum of FB1, FB2, FB3 and FB4. Horizontal line spanning all groups represents the mean response of all observations depicted within the panel. Red boxes represent quantiles, and blue lines represent means and standard deviations. Means not sharing the same letters above the plots are significantly different as determined by Tukey’s honestly significant difference (HSD) tests (P<0.05). No significant difference between strain total fumonisin level means are indicated as “nds” (P>0.05). Independent tests were performed for individual temperatures and days.

## Supplementary Figures


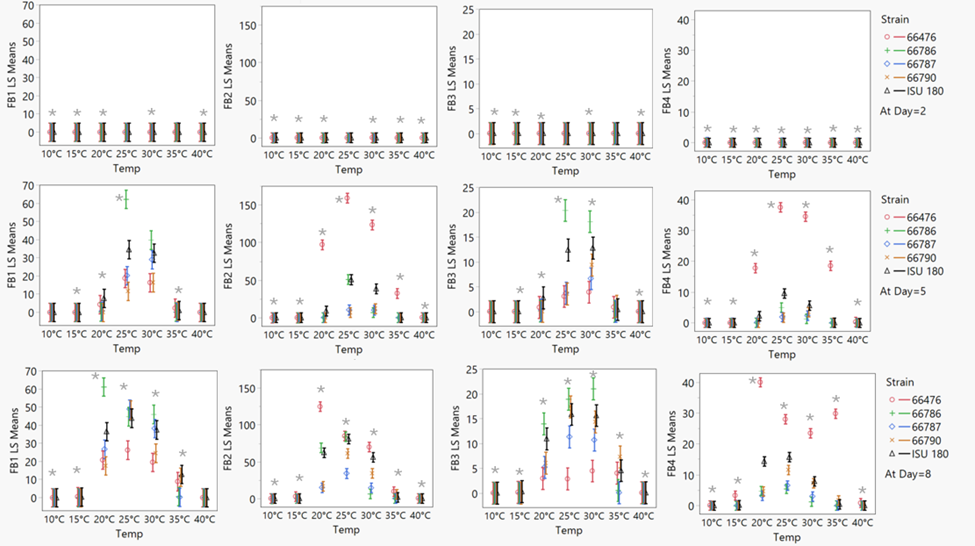


**Supplementary Figure 3.** Least squares (LS) means plots for fumonisin FB1 – FB4 as estimated by full factorial (5x7x3; 5 strains,7 temperatures, 3 time points), fit least squares means statistical analysis for individual F. verticillioides strains at different temperatures by timepoint (day). Bars represent confidence limits. Gray * denotes temperatures for which strain specific significant differences were determined by Tukey’s honestly significant difference (HSD) tests (P<0.05).

# Supplementary Tables

## Supplementary Table 1.

# Table 1. *F. verticillioides* isolates used in study

| **Strain** | **Geographic_Origin** | **Substrate_Origin** | **Substrate_Origin_Latin** | **GPS Coordinates** |
| --- | --- | --- | --- | --- |
| ISU 180 | USA: Iowa | Maize kernel | *Zea mays* | 42.0308° N, 93.6319° W |
| ISU 18B | USA: Iowa | Maize kernel | *Zea mays* | 42.0308° N, 93.6319° W |
| ISU 80 | USA: Iowa | Maize kernel | *Zea mays* | 42.0308° N, 93.6319° W |
| NRRL 20955 (FRC M-3120) | USA: California: San Joaquin County | Sorghum | *Sorghum bicolor* | 37.9176° N, 121.1710° W |
| FGSC 7600 (= NRRL 20956 = FRC M-3125) | USA: California: Visalia | Maize kernel | *Zea mays* | 36.3301° N, 119.2966° W |
| NRRL 20960 (= MRC-826) | South Africa: Transkei | Maize | *Zea mays* | 31.4632° S, 29.2321° E |
| NRRL 20984 (= FGSC 7603) | USA: Indiana: Knightstown | Maize kernel | *Zea mays* | 39.7956° N, 85.5264° W |
| NRRL 22055 (= FRC M-5500) | Nepal: Kathmandu | Maize kernel | *Zea mays* | 27.7103° N, 85.3222° E |
| NRRL 22056 (= FRC M-5538) | Nepal: Kathmandu | Maize kernel | *Zea mays* | 27.7103° N, 85.3222° E |
| NRRL 25457 | USA: Georgia: Savannah | Maize kernel | *Zea mays* | 32.0809° N, 81.0912° W |
| NRRL 66476 (= AMR F-7) | USA: Illinois | Maize kernel | *Zea mays* | 40.6927° N, 89.5928° W |
| NRRL 66477 (= AMR F-12) | USA: Illinois | Maize kernel | *Zea mays* | 40.6927° N, 89.5928° W |
| NRRL 66784 (= 8083a) | Mexico | Teosinte | *Zea mays mexicana* | 19.4326° N, 99.1332° W |
| NRRL 66785 (= NEP 833) | Nepal | Millet | Poaceae | 27.7103° N, 85.3222° E |
| NRRL 66786 (= NEP 877) | Nepal | Rice | *Oryza sativa* | 27.7103° N, 85.3222° E |
| NRRL 66787 (= 566692) | Michoacan Mexico | Teosinte | *Zea mays mexicana* | 19.5665° N, 101.7068° W |
| NRRL 66788 (= Fv152) | USA: Illinois | Maize | *Zea mays* | 40.1163° N, 88.2435° W |
| NRRL 66789 (= BP41-109) | USA: Oklahoma: Stillwater | Wheat | *Triticum* sp. | 36.1156° N, 97.0584° W |
| NRRL 66790 (= GGM 9) | Nepal | Maize | *Zea mays* | 27.7103° N, 85.3222° E |

## Supplementary Table 2.

# Table 2. 52 housekeeping gene sequences used for maximum likelihood tree inference.

| **Gene ID** | **Gene Model** | **GenBank Accession** | **Chromosome** | **Function** |
| --- | --- | --- | --- | --- |
| AAT1 | FVEG_06592 | XP_018752152.1 | 7 | Amino acid transporter |
| ACL1 | FVEG_04667 | XP_018749212.1 | 4 | ATP citrate lyase large subunit |
| ACT1 | FVEG_00630 | XP_018742925.1 | 1 | Actin |
| ALA1 | FVEG_00675 | XP_018742981.1 | 1 | alanyl-tRNA synthetase |
| ARO7 | FVEG_15416 | XP_018748478.1 | 2 | chorismate mutase |
| CAL1 | FVEG_07362 | XP_018753358.1 | 8 | Calmodulin |
| CAR1 | FVEG_10718 | XP_018758043.1 | 11 | Carotenoid biosynthesis |
| CDC60 | FVEG_04639 | XP_018749163.1 | 4 | leucyl-tRNA synthetase, cytoplasmic |
| COP1 | FVEG_12136 | XP_018759968.1 | 4 | coatomer protein complex, subunit alpha (xenin) |
| CPA1 | FVEG_07014 | XP_018752775.1 | 7 | Sodium/hydrogen exchanger subfamily CPA1-like family monovalent cation:H+ antiporter |
| CPR1 | FVEG_06400 | XP_018751900.1 | 2 | Cytochrome P450 Reductase |
| DPA1 | FVEG_16016 | XP_018752950.1 | 7 | DNA polymerase Alpha |
| DPD1 | FVEG_07987 | XP_018754251.1 | 3 | DNA polymerase Delta |
| DPE1 | FVEG_04699 | XP_018749259.1 | 4 | DNA polymerase Epsilon |
| ERG1 | FVEG_06181 | XP_018751552.1 | 2 | Ergosterol Monooxygenase/Oxidase |
| FAS1 | FVEG_04241 | XP_018748624.1 | 2 | Fatty Acid Synthase Alpha Subunit |
| FAS2 | FVEG_04242 | XP_018748627.1 | 2 | Fatty Acid Synthase Beta Subunit |
| FLB1 | FVEG_08855 | XP_018755471.1 | 10 | Regulatory Gene |
| FPS1 | FVEG_05204 | XP_018750151.1 | 3 | Farnesyl Pyrrophosphate Synthase |
| GPD1 | FVEG_04927 | XP_018749661.1 | 4 | Glyceraldehyde 3-Phosphate Dehydrogenase |
| HGR1 | FVEG_03711 | XP_018747829.1 | 2 | Hydroxy Methyl gluteryl CoA Reductase |
| HIS3 | FVEG_11374 | XP_018758918.1 | 9 | Histone H3 |
| HSH155 | FVEG_08126 | XP_018754502.1 | 3 | U2 snRNP spliceosome subunit |
| KU70 | FVEG_04235 | XP_018748618.1 | 2 | Nonhomologous DNA break repair |
| LAE1 | FVEG_00539 | XP_018742754.1 | 1 | Global Regulatory Gene/Chromatin Methyl Transferase |
| LCB1 | FVEG_12143 | XP_018759981.1 | 4 | Sphinganine Palmitoyl Transferase Subunit 1 |
| LCB2 | FVEG_10287 | XP_018757422.1 | 9 | Sphinganine Palmitoyl Transferase Subunit 2 |
| LRO1 | FVEG_05051 | XP_018749872.1 | 4 | Phospholipid:diacylglycerol acyltransferase |
| LTE1 | FVEG_11583 | XP_018759273.1 | 7 | Guanine nucleotide exchange factor LTE1 |
| MCM7 | FVEG_08063 | XP_018754394.1 | 3 | Replication Licensing Factor |
| PGK1 | FVEG_10153 | XP_018757237.1 | 9 | Phosphoglycerate Kinase |
| PHO5 | FVEG_12069 | XP_018759883.1 | 4 | Phosphate Permease |
| PPT1 | FVEG_01894 | XP_018744954.1 | 6 | 4'-Phosphopantetheinyl Transferase |
| RED1 | FVEG_13368 | XP_018761549.1 | 8 | Reductase |
| RPA1 | FVEG_11373 | XP_018758914.1 | 9 | DNA-directed RNA polymerase I subunit RPA1 |
| RPA2 | FVEG_06860 | XP_018752517.1 | 7 | DNA-directed RNA polymerase I subunit RPA2 |
| RPB1 | FVEG_00683 | XP_018742997.1 | 1 | RNA Polymerase Largest Subunit |
| RPB2 | FVEG_09286 | XP_018756128.1 | 5 | RNA Polymerase 2nd Largest Subunit |
| RPC2 | FVEG_00714 | XP_018743044.1 | 1 | DNA-directed RNA polymerase III subunit RPC2 |
| RVB1 | FVEG_04821 | XP_018749465.1 | 4 | RuvB-like helicase 1 |
| SPH1 | FVEG_06971 | XP_018752696.1 | 7 | Sphinganine N-Acyl Transferase Subunit 1 |
| SPH2 | FVEG_12887 | XP_018760957.1 | 11 | Sphinganine N-Acyl Transferase Subunit 2 |
| TEF1 | FVEG_02381 | XP_018745816.1 | 6 | Translation Elongation Factor 1-alpha |
| TFB4 | FVEG_11534 | XP_018759190.1 | 7 | transcription initiation factor TFIIH subunit 3 |
| TOP1 | FVEG_05113 | XP_018749972.1 | 3 | Topoisomerase |
| TOR2 | FVEG_01648 | XP_018744630.1 | 6 | FKBP12-rapamycin complex-associated protein |
| TPS1 | FVEG_04683 | XP_018749233.1 | 4 | Trehalose Phosphate Synthase |
| TRI101 | FVEG_00056 | XP_018742047.1 | 1 | Trichothecene-3-acetyl transferase |
| TSR1 | FVEG_11260 | XP_018758717.1 | 9 | Ribosomal Biogenesis Protein |
| TUB1 | FVEG_00557 | XP_018742806.1 | 1 | Tubulin alpha subunit |
| TUB2 | FVEG_04081 | XP_018748359.1 | 2 | Tubulin beta subunit |
| UBT1 | FVEG_01360 | XP_018744176.1 | 1 | Ubiqitin Thioesterase |
